# Supplementary material for: Price negotiation and pricing of anticancer drugs in China: An observational study
Source: PLoS Med. 2024 Jan 2;21(1):e1004332. doi: 10.1371/journal.pmed.1004332 (PMC10793910; doi:10.1371/journal.pmed.1004332)
Supplement: S1 Text — (DOCX) [file pmed.1004332.s001.docx]

**S1 Text. Price negotiation in China**

In China, the National Healthcare Security Administration (NHSA) is currently responsible for price negotiation and reimbursement decisions. To address the issue of high drug prices, promote patient access, and ensure the sustainability of the medical insurance fund, China formally launched national reimbursement-linked price negotiation in 2017 [1]. As of January 2023, the NHSA has conducted six rounds of price negotiations directly with pharmaceutical companies, resulting in the inclusion of numerous newly negotiated drugs in the NRDL (Table 1).

**Table 1. Outcomes of price negotiations in China from 2017 to 2022**

| Year of negotiation | Drugs to be negotiated | Drugs negotiated successfully | | | Anticancer drugs negotiated successfully | | |
| --- | --- | --- | --- | --- | --- | --- | --- |
|  |  | Total | New additions | Re-negotiations | Total | New additions | Re-negotiations |
| 2017 | 44 | 36 | 36 | 0 | 15 | 15 | 0 |
| 2018 | 18 | 17 | 17 | 0 | 16 | 16 | 0 |
| 2019 | 150 | 97 | 70 | 27 | 18 | 8 | 9 |
| 2020 | 162 | 119 | 96 | 23 | 30 | 14 | 16 |
| 2021 | 117 | 94 | 67 | 27 | 38 | 18 | 20 |
| 2022 | 147 | 121 | 108 | 13 | 22 | 13 | 9 |

Note: Authors’ analysis of data from the NHSA. We did not include traditional Chinese medicines for the treatment of cancer in this analysis.

The process of price negotiation over the years is generally similar and can be roughly divided into several stages: the preparation stage, application stage, expert review stage, negotiation stage, and results announcement stage [1,2]. Using the year 2022 as an example [3], during the preparation stage, the NHSA released a working plan and guidelines and convened expert committees for price negotiation. In the application stage, pharmaceutical companies would file for negotiation to obtain reimbursement for drugs. Subsequently, the NHSA conducted an initial eligibility screening, announcing candidate drugs under consideration for reimbursement based on the screening results.

At the review stage, expert committees assessed whether drugs were suitable for price negotiation or other pathways through joint reviews. For drugs eligible for price negotiation, experts determined drug specifications, reference drugs, and reimbursement conditions. During the negotiation stage, pharmaceutical companies submitted dossiers for evaluation by experts. The dossiers included information on drug safety, efficacy, economic evaluation, innovativeness, and equity. The NHSA's target prices were determined by two parallel groups: public medical insurance executives and pharmacoeconomics experts. The former estimated prices based on pre-negotiation prices and the sustainability of the insurance fund. Meanwhile, the latter focused on the comparative effectiveness and safety of the candidates compared to existing treatments. They assessed pharmacoeconomic reports and budget impact analyses submitted by pharmaceutical companies, using domestic and international prices as references, in order to align prices more closely with clinical benefits.

The NHSA then conducted price negotiations with pharmaceutical companies, and the agreed-upon price was established. During the results announcement stage, the NHSA released the updated version of the NRDL, which included drugs that had undergone successful price negotiations. Drugs listed in the NRDL were reimbursed by the NHSA.

References

1. Liu GG, Wu J, He X, Jiang Y. Policy Updates on Access to and Affordability of Innovative Medicines in China. Value Health Reg Issues*.* 2022;30:59-66
2. Wen J, Li M, Jiang Y. Cost effectiveness of innovative anti-cancer drugs and reimbursement decisions in China. Health Policy Technol. 2023;12(2):100742
3. National Healthcare Security Administration (NHSA). Announcement on the release of the '2022 National Basic Medical Insurance, Work Injury Insurance, and Maternity Insurance Drug Catalog Adjustment Work Plan' and associated documents. 2022 [Accessed 2023 May 01]. http://www.nhsa.gov.cn/art/2022/6/29/art_109_8342.html
