# Supplementary material for: Price negotiation and pricing of anticancer drugs in China: An observational study
Source: PLoS Med. 2024 Jan 2;21(1):e1004332. doi: 10.1371/journal.pmed.1004332 (PMC10793910; doi:10.1371/journal.pmed.1004332)
Supplement: S2 Text — (DOCX) [file pmed.1004332.s002.docx]

**S2 Text. Regression equations of models**

1. Model (1) and (2) in Table 2 and Table 3.

$${Costs}_{i}=\beta_{0}+\boldsymbol{value}_{\boldsymbol{i}}^{\boldsymbol{‘}}\boldsymbol{\beta}+\boldsymbol{x}_{\boldsymbol{i}}^{\boldsymbol{'}}\boldsymbol{\gamma}+\varepsilon_{i}$$

Notes: ${Costs}_{i}$represents the treatment costs over the expected treatment duration of therapeutic indication *i*. In the model (1) of Table 2 and Table 3, ${Costs}_{i}$ represents the treatment costs before negotiation, while in the model (2) of Table 2 and Table 3, ${Costs}_{i}$ represents the treatment costs after negotiation. $\boldsymbol{value}_{\boldsymbol{i}}$ represents the vector of different measures of clinical value for anticancer drugs. For therapeutic indication *i* supported by randomized controlled trials, measures of clinical value include survival benefits, safety and quality of life (Table 2). For therapeutic indication *i* supported by single-arm clinical trials, the measure of clinical value is ORR (Table 3). $\boldsymbol{\beta}$ is the coefficients of interest. A positive value means that the clinical value of anticancer drugs is positively associated with treatment costs. $\boldsymbol{x}_{\boldsymbol{i}}$ represents the vector of control variables, including line of therapy, cancer site, baseline survival, domestically developed status, year of approval, priority review, conditional approval, blind method, comparator and administration route.

1. Model (3) in Table 2 and Table 3.

$${Costs}_{i}=\beta_{0}+{\boldsymbol{v}\boldsymbol{alue}}_{\boldsymbol{i}}^{\boldsymbol{‘}}\boldsymbol{\beta}+\delta{negotiation}_{i}+\boldsymbol{\Delta\beta}^{'}\boldsymbol{value}_{\boldsymbol{i}}\cdot{negotiation}_{i}+\boldsymbol{x}_{\boldsymbol{i}}^{\boldsymbol{'}}\boldsymbol{\gamma}+\varepsilon_{i}$$

Notes: ${Costs}_{i}$ represents the treatment costs over the expected treatment duration of the therapeutic indication *i* before and after negotiation. $\boldsymbol{value}_{\boldsymbol{i}}$ represents the vector of different measures of clinical value for anticancer drugs. For therapeutic indication *i* supported by randomized controlled trials, measures of clinical value include survival benefits, safety and quality of life (Table 2). For therapeutic indication *i* supported by single-arm clinical trials, the measure of clinical value is ORR (Table 3). The binary variable *negotiation* stands for the status (before price negotiation or after price negotiation) of therapeutic indication *i*. To examine the impact of price negotiation on the association between treatment costs and clinical value, the interaction term of negotiation and clinical value is added and is represented by $\boldsymbol{value}_{\boldsymbol{i}}\cdot{negotiation}_{i}$. $\boldsymbol{\Delta\beta}$represents the parameters of interest, and a significant value indicates that the relationship between treatment costs and clinical value is mediated by price negotiation. $\boldsymbol{x}_{\boldsymbol{i}}$ represents the vector of control variables, including line of therapy, cancer site, baseline survival, domestically developed status, year of approval, priority review, conditional approval, blind method, comparator and administration route.
