# Supplementary material for: Price negotiation and pricing of anticancer drugs in China: An observational study
Source: PLoS Med. 2024 Jan 2;21(1):e1004332. doi: 10.1371/journal.pmed.1004332 (PMC10793910; doi:10.1371/journal.pmed.1004332)
Supplement: S3 Text — (DOCX) [file pmed.1004332.s003.docx]

**S3 Text. Sensitivity analyses**

We performed several sensitivity checks to ensure the robustness of our findings. First, we excluded indications for anticancer drugs launched before price negotiation (i.e., 2017), to account for possible bias due to the longer time lag between market authorization and price negotiation. Second, we re-estimated regression models for indications supported by randomized controlled trials, using survival benefits in overall survival (OS) and survival benefits in progression-free survival (PFS) as separate survival measures, instead of aggregated survival benefits in either OS or PFS.
