# Supplementary material for: Price negotiation and pricing of anticancer drugs in China: An observational study
Source: PLoS Med. 2024 Jan 2;21(1):e1004332. doi: 10.1371/journal.pmed.1004332 (PMC10793910; doi:10.1371/journal.pmed.1004332)
Supplement: S1 Table — (DOCX) [file pmed.1004332.s004.docx]

**S1 Table. Overview of variables**

| **Variables** | **Definition and explanation** | **Operationalization** | **Data Source** |
| --- | --- | --- | --- |
| *Dependent variables* | | | |
| Costs before negotiation | Treatment costs over expected treatment durations on the grounds of pre-negotiation prices | Numeric | The MOHRSS, the NHSA, and the Drugdataexpy |
| Costs after negotiation | Treatment costs over expected treatment durations on the grounds of negotiated reimbursement prices | Numeric | The Drugdataexpy |
| *Explanatory variables* | | | |
| Survival benefits | Survival benefits in overall survival (OS) or progression-free survival (PFS) of the study drug in relation to its comparator expressed in months | Numeric | Publications of pivotal clinical trials |
| Quality of life | The comparable quality of life of the study drug in relation to its comparator | Categorical: 0=no difference, 1=improvement, 2=reduction or not available | Publications of pivotal clinical trials |
| Safety | The comparable safety of the study drug in relation to its comparator | Categorical: 0=reduction, 1=improvement or no difference | Publications of pivotal clinical trials |
| Overall response rate | Overall response rate of the indications supported by single-arm clinical trials | Numeric | Publications of pivotal clinical trials |
| *Control variables* | | | |
| Line of therapy | The order in which different therapies are given to people as their disease progresses. | Categorical: 0=other lines, 1=first-line | Drug labels or clinical trials |
| Cancer site | Disease area | Categorical: 0=blood, 1=lung, 2=breast, 3=colorectal, 4=renal, and 5=other for indications supported by randomized controlled trials.  0=hematological, and 1= non- hematological for indications supported by single-arm clinical trials. | Drug labels |
| Domestically developed | Whether the drug was developed in China or imported from abroad. | Categorical: 0=imported, 1=domestic. | Drug labels |
| Year of approval | The year of the therapeutic indication approved | Categorical: 0=before 2017, 1= 2017 and beyond | CDE, NMPA |
| Priority review | Market authorization pathway that expedites the review process for drugs | Categorical: 0=non-priority review, 1=priority review | CDE, NMPA |
| Conditional approval | Conditional marketing authorization for drugs | Categorical: 0=non-conditional, 1= conditional | CDE, NMPA |
| Intravenous | Route of administration. Oral drugs are more convenient for patients than physician-administered intravenous drugs. Intravenous-administered drugs are not preferred by patients, and are associated with more work for physicians, potentially decreasing their value | Categorical: 0=oral, 1= intravenous | Drug labels |
| Baseline survival | Survival in the control arm of pivotal studies | Numeric | Publications of pivotal clinical trials |
| Comparator | The reference drug chose in randomized clinical trial of the study anticancer drug. Higher quality of clinical data available at price negotiation is associated with less uncertainty about a drug’s clinical value and may, therefore, increase its value | Categorical: 0=placebo, 1= active | Publications of pivotal clinical trials |
| Blind | The blind method used in clinical trials | Categorical: 0=no, 1= yes | Publications of pivotal clinical trials |
| Notes: MOHRSS: the Ministry of Human Resources and Social Security; NHSA, National Healthcare Security Administration; CDE, NMPA, Center for Drug Evaluation, National Medical Products Administration. | | | |
