# Supplementary material for: Price negotiation and pricing of anticancer drugs in China: An observational study
Source: PLoS Med. 2024 Jan 2;21(1):e1004332. doi: 10.1371/journal.pmed.1004332 (PMC10793910; doi:10.1371/journal.pmed.1004332)
Supplement: S2 Table — (DOCX) [file pmed.1004332.s005.docx]

**S2 Table. Information on 76 indications of anticancer drugs supported by randomized controlled trials in China.**

| **No.** | **Generic name** | **Indication** | **Year of negotiation** | **Clinical trial** | **Comparator (s)** | **Costs before negotiation (US$)** | **Costs after negotiation (US$)** | **Survival benefits (months)** | **Quality of life** | **Safety** | **Cancer site** | **First-line** | **Domestically developed** | **Priority review** | **Administration route** | **Blind** | **Year of indication approval** |
| --- | --- | --- | --- | --- | --- | --- | --- | --- | --- | --- | --- | --- | --- | --- | --- | --- | --- |
| 1 | Abiraterone | metastatic castration-resistant prostate cancer | 2017 | NCT00638690 | placebo plus prednisone | 43,705 | 20,583 | 4.6 | improvement | no difference | prostate | no | imported | no | oral | yes | 2015 |
| 2 | Apatinib | advanced or metastatic sdenocarcinoma of the stomach or gastroesophageal junction | 2017 | not available | placebo | 7,800 | 4,905 | 1.8 | not available | reduction | gastric & esophageal | no | domestic | no | oral | no | 2014 |
| 3 | Bevacizumab | metastatic colorectal cancer | 2017 | NCT00109070 | placebo plus standard chemotherapy | 52,183 | 20,143 | 4.7 | no difference | reduction | colorectal | yes | imported | no | intravenous | yes | 2010 |
| 4 | Bevacizumab | advanced non-squamous NSCLC | 2017 | NCT01364012 | carboplatin/paclitaxel plus placebo | 34,461 | 13,302 | 6.6 | not available | reduction | lung | yes | imported | no | intravenous | yes | 2015 |
| 5 | Erlotinib | EGFR mutation-positive advanced NSCLC (maintenance treatment) | 2017 | NCT00556712 | placebo | 5,860 | 2,484 | 0.2 | no difference | reduction | lung | no | imported | no | oral | yes | 2017 |
| 6 | Erlotinib | EGFR mutation-positive advanced NSCLC (previously treated) | 2017 | NCT00026325 | placebo | 4,492 | 1,904 | 2.0 | improvement | reduction | lung | no | imported | no | oral | yes | 2006 |
| 7 | Erlotinib | EGFR mutation-positive advanced NSCLC (first-line) | 2017 | NCT01342965 | gemcitabine plus cisplatin | 16,742 | 7,097 | 0.8 | not available | improvement | lung | yes | imported | no | oral | no | 2017 |
| 8 | Lapatinib | HER-2-positive advanced breast cancer | 2017 | not available | capecitabine | 27,016 | 15,949 | 2.4 | not available | reduction | breast | no | imported | no | oral | no | 2013 |
| 9 | Lenalidide | multiple myeloma | 2017 | NCT00056160 | placobo plus dexamethasone | 102,934 | 40,719 | 9.4 | not available | reduction | haematological | no | imported | no | oral | yes | 2013 |
| 10 | Rituximab | CD20 positive advanced follicular non-Hodgkin lymphoma | 2017 | NCT00004179 | cyclophosphamide, doxorubicin hydrochloride, prednisone, vincristine sulfate | 11,515 | 6,337 | 12.9 | not available | reduction | haematological | no | imported | no | intravenous | no | not available |
| 11 | Rituximab | Diffuse Large-B-Cell Lymphoma | 2017 | not available | cyclophosphamide, doxorubicin, vincristine, and prednisone | 23,029 | 12,674 | 58.8 | not available | reduction | haematological | no | imported | no | intravenous | no | not available |
| 12 | Nimotuzumab | EGFR mutation-positive advanced nasopharyngeal cancer | 2017 | not available | radiotherapy | 5,601 | 4,024 | 3.6 | not available | reduction | head & neck | yes | domestic | no | intravenous | no | 2008 |
| 13 | Bortezomib | multiple myeloma (first-line) | 2017 | NCT00111319 | melphalan/prednisone | 18,353 | 8,324 | 13.3 | no difference | reduction | haematological | yes | imported | no | intravenous | no | not available |
| 14 | Bortezomib | relapsed multiple myeloma | 2017 | NCT00048230 | dexamethasone | 36,706 | 16,648 | 6.1 | not available | reduction | haematological | no | imported | no | intravenous | no | not available |
| 15 | Trastuzumab | HER2 positive metastatic breast cancer | 2017 | not available | chemotherapy | 43,260 | 14,618 | 4.8 | not available | reduction | breast | yes | imported | no | intravenous | no | not available |
| 16 | Trastuzumab | HER2-positive advanced gastric or gastro-oesophageal junction cancer | 2017 | NCT01041404 | capecitabine plus cisplatin or fluorouracil plus cisplatin | 26,622 | 8,995 | 2.7 | improvement | no difference | breast | yes | imported | no | intravenous | no | not available |
| 17 | Sorafenib | advanced hepatocellular carcinoma | 2017 | NCT00492752 | placebo | 19,536 | 9,551 | 2.3 | no difference | reduction | hepatobiliary | yes | imported | no | oral | yes | not available |
| 18 | Sorafenib | renal-cell carcinoma | 2017 | NCT00073307 | placebo | 19,782 | 9,671 | 2.6 | no difference | reduction | renal | no | imported | no | oral | yes | not available |
| 19 | Sorafenib | thyroid cancer | 2017 | NCT00984282 | placebo | 39,072 | 19,102 | 5.0 | not available | reduction | Thyroid | yes | imported | no | oral | yes | not available |
| 20 | Everolimus | advanced pancreatic neuroendocrine tumors | 2017 | NCT00510068 | placebo plus best supportive care | 19,942 | 11,925 | 6.3 | not available | reduction | neuroendocrine | yes | imported | no | oral | yes | 2014 |
| 21 | Everolimus | advanced renal cell carcinoma | 2017 | NCT00410124 | best supportive care plus placebo | 10,326 | 6,175 | 0.4 | no difference | reduction | renal | no | imported | no | oral | yes | 2013 |
| 22 | Recombinant Human Endostatin Injection | advanced NSCLC | 2017 | not available | placebo plus vinorelbine and cisplatin | 3,144 | 2,423 | 5.0 | improvement | reduction | lung | yes | domestic | no | intravenous | yes | 2006 |
| 23 | Afatinib | advanced or metastatic NSCLC (squamous) | 2018 | NCT01523587 | erlotinib | 6,009 | 3,658 | 1.1 | improvement | reduction | lung | no | imported | yes | oral | no | 2017 |
| 24 | Afatinib | advanced or metastatic NSCLC with EGFR mutations | 2018 | NCT00949650 | cisplatin and pemetrexed | 16,389 | 9,976 | 0.0 | improvement | reduction | lung | yes | imported | yes | oral | no | 2017 |
| 25 | Axitinib | advanced RCC | 2018 | NCT00678392 | sorafenib | 52,387 | 15,394 | 0.9 | no difference | reduction | renal | no | imported | no | oral | no | 2015 |
| 26 | Azacitidine | myelodysplastic syndromes | 2018 | NCT00071799 | conventional care | 49,992 | 20,092 | 9.5 | not available | reduction | haematological | yes | imported | yes | intravenous | no | 2017 |
| 27 | Azacitidine | acute myeloid leukemia | 2018 | NCT01074047 | conventional care | 33,328 | 13,395 | 3.9 | no difference | improvement | haematological | yes | imported | yes | intravenous | no | 2017 |
| 28 | Anlotinib | advanced or metastatic NSCLC | 2018 | NCT02388919 | placebo | 11,245 | 6,183 | 3.3 | improvement | reduction | lung | no | domestic | yes | oral | yes | 2018 |
| 29 | Osimertinib | EGFR T790M advanced NSCLC | 2018 | NCT02151981 | pemetrexed plus either carboplatin or cisplatin | 103,875 | 31,914 | 5.7 | improvement | improvement | lung | no | imported | yes | oral | no | 2017 |
| 30 | Crizotinib | LK-Positive Advanced NSCLC | 2018 | NCT01639001 | pemetrexed plus either carboplatin or cisplatin | 118,704 | 34,661 | 0.8 | improvement | reduction | lung | yes | imported | yes | oral | no | 2013 |
| 31 | Pazopanib | advanced RCC | 2018 | NCT00334282 | placebo | 52,346 | 21,475 | 2.4 | no difference | reduction | renal | yes | imported | no | oral | yes | 2017 |
| 32 | Regorafenib | metastatic colorectal cancer | 2018 | NCT01103323 | best supportive care plus placebo | 13,712 | 7,466 | 1.4 | no difference | reduction | colorectal | no | imported | yes | oral | yes | 2017 |
| 33 | Regorafenib | advanced gastrointestinal stromal tumours | 2018 | NCT01271712 | placebo | 26,167 | 14,247 | 3.9 | no difference | reduction | gist | no | imported | yes | oral | yes | 2017 |
| 34 | Regorafenib | hepatocellular carcinoma | 2018 | NCT01774344 | placebo | 38,198 | 20,797 | 2.8 | no difference | reduction | hepatobiliary | no | imported | no | oral | yes | 2017 |
| 35 | Ceritinib | ALK mutation-positive advanced or metastatic NSCLC | 2018 | NCT01828112 | chemotherapy (pemetrexed or docetaxel） | 72,036 | 19,043 | -2.0 | improvement | reduction | lung | no | imported | yes | oral | no | 2018 |
| 36 | Sunitinib | advanced gastrointestinal stromal tumours | 2018 | NCT00075218 | placebo | 130,176 | 46,050 | 1.8 | not available | reduction | gist | no | imported | no | oral | yes | not available |
| 37 | Sunitinib | advanced pancreatic neuroendocrine tumors | 2018 | NCT00428597 | placebo | 27,418 | 9,699 | 9.5 | no difference | reduction | neuroendocrine | yes | imported | no | oral | yes | not available |
| 38 | Sunitinib | metastatic RCC | 2018 | NCT00083889 | interferon alfa | 58,280 | 20,617 | 4.6 | improvement | reduction | renal | yes | imported | no | oral | no | not available |
| 39 | Vemurafenib | BRAFV600 mutation-positive unresectable or metastatic melanoma | 2018 | NCT01006980 | dacarbazine | 44,293 | 26,815 | 3.9 | not available | reduction | melanoma（skin） | yes | imported | yes | oral | no | 2017 |
| 40 | Cetuximab | metastatic colorectal cancer | 2018 | NCT00063141;NCT00065598 | irinotecan | 29,665 | 11,549 | 0.7 | improvement | reduction | colorectal | no | imported | no | intravenous | no | not available |
| 41 | Ibrutinib | mantle-cell lymphoma | 2018 | NCT01646021 | temsirolimus | 141,040 | 49,364 | 6.8 | not available | improvement | haematological | no | imported | yes | oral | no | 2017 |
| 42 | Ibrutinib | chronic lymphocytic leukemia or small lymphocytic lymphoma | 2018 | NCT01578707 | ofatumumab | 301,179 | 105,413 | 36.0 | improvement | reduction | haematological | no | imported | yes | oral | no | 2017 |
| 43 | Ixazomib | multiple myeloma | 2018 | NCT01564537 | placebo plus lenalidomide and dexamethasone | 75,091 | 40,263 | 5.9 | no difference | reduction | haematological | no | imported | yes | oral | yes | 2018 |
| 44 | Alectinib | advanced ALK-positive NSCLC | 2019 | NCT02075840 | crizotinib | 108,914 | 33,193 | 23.9 | not available | improvement | lung | yes | imported | yes | oral | no | 2018 |
| 45 | Olaparib | platinum-sensitive, relapsed ovarian cancer or primary peritoneal cancer | 2019 | NCT01874353 | placebo | 223,775 | 51,561 | 12.9 | no difference | reduction | ovarian | no | imported | yes | oral | yes | 2018 |
| 46 | Pyrotinib | HER2 positive metastatic breast cancer | 2019 | NCT03080805. | lapatinib plus capecitabine | 34,935 | 13,689 | 5.7 | not available | reduction | breast | no | domestic | yes | oral | no | 2018 |
| 47 | Fruquintinib | metastatic colorectal cancer | 2019 | NCT02314819 | placebo plus best supportive care | 11,762 | 4,252 | 2.7 | not available | reduction | colorectal | no | domestic | yes | oral | yes | 2018 |
| 48 | Raltitrexed | metastatic colorectal cancer | 2019 | not available | 5-fluorouracil plus leucovorin | 8,711 | 3,486 | 0.0 | reduction | improvement | colorectal | no | domestic | no | intravenous | yes | 2009 |
| 49 | Dabrafenib | BRAFV600 mutation-positive unresectable or metastatic melanoma | 2020 | NCT01597908 | vemurafenib | 60,096 | 16,064 | 8.2 | improvement | improvement | melanoma（skin） | yes | imported | yes | oral | no | 2019 |
| 50 | Enzalutamide | metastatic castration-resistant prostate cancer | 2020 | NCT01212991 | placebo | 101,725 | 22,027 | 4.0 | improvement | reduction | prostate | no | imported | yes | oral | yes | 2019 |
| 51 | Camrelizumab | advanced or metastatic non-squamousNSCLC | 2020 | NCT03134872 | carboplatin and pemetrexed | 31,561 | 4,667 | 7.4 | not available | reduction | lung | yes | domestic | yes | intravenous | no | 2020 |
| 52 | Camrelizumab | advanced or metastatic oesophageal squamous cell carcinoma | 2020 | NCT03099382 | chemotherapy with docetaxel or irinotecan | 17,215 | 2,546 | 2.1 | improvement | improvement | oesophageal | no | domestic | yes | intravenous | no | 2020 |
| 53 | Lenvatinib | unresectable hepatocellular carcinoma | 2020 | NCT01761266 | sorafenib | 41,629 | 8,028 | 1.3 | no difference | reduction | hepatobiliary | yes | imported | yes | oral | no | 2018 |
| 54 | Niraparib | Platinum-sensitive, recurrent ovarian cancer | 2020 | NCT01847274 | placebo | 228,137 | 54,775 | 15.5 | no difference | reduction | ovarian | no | domestic | yes | oral | yes | 2019 |
| 55 | Trametinib | BRAFV600 mutation-positive unresectable or metastatic melanoma | 2020 | NCT01584648 | dabrafenib and placebo | 80,128 | 32,126 | 6.4 | improvement | no difference | melanoma（skin） | yes | imported | yes | oral | yes | 2019 |
| 56 | Inetetamab | HER2 positive metastatic breast cancer | 2020 | not available | vinorelbine | 21,284 | 8,208 | 5.9 | not available | reduction | breast | no | domestic | yes | intravenous | no | 2020 |
| 57 | Abemaciclib | advanced or metastatic breast cancer (plus aromatase inhibitor) | 2021 | NCT02246621 | placebo plus either anastrozole or letrozole | 45,666 | 14,024 | 13.4 | no difference | reduction | breast | yes | imported | yes | oral | yes | 2020 |
| 58 | Abemaciclib | advanced or metastatic breast cancer (plus fulvestrant) | 2021 | NCT02107703 | placebo plus fulvestrant | 45,426 | 13,950 | 9.4 | no difference | reduction | breast | no | imported | yes | oral | yes | 2020 |
| 59 | Apalutamide | metastatic castration-resistant prostate cancer | 2021 | NCT01946204 | placebo | 250,574 | 41,448 | 14.0 | no difference | reduction | prostate | no | imported | yes | oral | yes | 2020 |
| 60 | Eribulin | metastatic breast cancer | 2021 | NCT00388726 | treatment of physician's choice | 20,630 | 3,763 | 2.7 | not available | reduction | breast | no | imported | no | oral | no | 2019 |
| 61 | Dacomitinib | advanced or metastatic NSCLC | 2021 | NCT01774721 | gefitinib | 13,516 | 2,224 | 7.1 | reduction | reduction | lung | yes | domestic | yes | oral | no | 2019 |
| 62 | Daratumumab | multiple myeloma (plus lenalidomide and dexamethasone) | 2021 | NCT02076009 | lenalidomide and dexamethasone | 157,603 | 91,526 | 27.0 | no difference | reduction | haematological | no | imported | yes | intravenous | no | 2021 |
| 63 | Daratumumab | multiple myeloma (plus bortezomib and dexamethasone) | 2021 | NCT02136134 | bortezomib and dexamethasone | 75,009 | 43,560 | 9.6 | no difference | reduction | haematological | no | imported | yes | intravenous | no | 2021 |
| 64 | Darolutamide | nonmetastatic castration-resistant prostate cancer | 2021 | NCT02200614 | placebo | 54,161 | 17,240 | 22.0 | improvement | reduction | prostate | no | imported | yes | oral | yes | 2021 |
| 65 | Donafenib | unresectable hepatocellular carcinoma | 2021 | NCT02645981 | sorafenib | 14,099 | 4,421 | 1.8 | not available | improvement | hepatobiliary | yes | domestic | yes | oral | yes | 2021 |
| 66 | Fluzoparib | platinum-sensitive, recurrent ovarian carcinoma | 2021 | NCT03863860 | placebo | 20,839 | 11,860 | 7.4 | no difference | reduction | ovarian | no | domestic | yes | oral | yes | 2021 |
| 67 | Pomalidomide | multiple myeloma | 2021 | NCT01311687 | dexamethasone | 10,420 | 3,595 | 4.6 | improvement | reduction | haematological | no | domestic | yes | oral | no | 2020 |
| 68 | Sulfatinib | extrapancreatic neuroendocrine tumours | 2021 | NCT02588170 | placebo | 20,053 | 9,603 | 5.4 | no difference | reduction | neuroendocrine | yes | domestic | yes | oral | yes | 2020 |
| 69 | Sulfatinib | pancreatic neuroendocrine tumours | 2021 | NCT02589821 | placebo | 21,559 | 10,312 | 7.2 | no difference | reduction | neuroendocrine | no | domestic | no | oral | yes | 2021 |
| 70 | Brigatinib | NSCLC | 2022 | NCT02737501 | crizotinib | 104,069 | 42,948 | 13 | improvement | reduction | lung | yes | imported | no | oral | no | 2022 |
| 71 | Trastuzumab Emtansine | advanced breast cancer | 2022 | NCT00829166 | capecitabine plus lapatinib | 33,132 | 14,222 | 4.0 | reduction | improvement | breast | no | imported | no | intravenous | no | 2020 |
| 72 | Ripretinib | gastrointestinal stromal tumours | 2022 | NCT03353753 | placebo | 37,354 | 14,501 | 8.5 | improvement | reduction | gist | no | imported | no | oral | yes | 2021 |
| 73 | Utidelone | breast cancer | 2022 | NCT02253459. | capecitabine | 13,181 | 4,043 | 3.8 | reduction | reduction | breast | no | domestic | yes | intravenous | no | 2021 |
| 74 | Brentuximab | T-cell lymphoma | 2022 | NCT01578499 | methotrexate or  bexarotene | 60,282 | 27,795 | 13.2 | no difference | improvement | haematological | no | imported | no | intravenous | no | 2020 |
| 75 | Venetoclax | acute myeloid leukemia | 2022 | NCT02993523 | azacitidine plus placebo | 54,363 | 23,319 | 5.1 | no difference | reduction | haematological | yes | imported | yes | intravenous | yes | 2020 |
| 76 | Dalpiciclib | breast cancer | 2022 | NCT03927456 | fulvestrant | 20,039 | 6,390 | 8.5 | reduction | reduction | breast | no | domestic | no | oral | yes | 2021 |
| Notes: NSCLC, non-small cell lung cancer. | | | | | |  |  |  |  |  |  |  |  |  |  |  |  |
