# Supplementary material for: Price negotiation and pricing of anticancer drugs in China: An observational study
Source: PLoS Med. 2024 Jan 2;21(1):e1004332. doi: 10.1371/journal.pmed.1004332 (PMC10793910; doi:10.1371/journal.pmed.1004332)
Supplement: S3 Table — (DOCX) [file pmed.1004332.s006.docx]

S3 Table. Information on 27 indications of anticancer drugs supported by single-arm clinical trials in China.

| **No.** | **Generic name** | **Indication** | **Year of negotiation** | **Clinical trial** | **Costs before negotiation (US$)** | **Costs after negotiation (US$)** | **ORR** | **Cancer site** | **First-line treatment** | **Domestically developed** | **Conditional approval** | **Administration route** | **Year of indication approval** |
| --- | --- | --- | --- | --- | --- | --- | --- | --- | --- | --- | --- | --- | --- |
| 1 | Bortezomib | relapsed or refractory mantle cell lymphoma | 2017 | NCT00063713 | 24,471 | 11,099 | 47.00% | hematological | no | imported | no | intravenous | not available |
| 2 | Chidamide/Tucidinostat | peripheral T-cell lymphoma | 2017 | not available | 8,792 | 6,152 | 28.00% | hematological | no | domestic | yes | oral | 2014 |
| 3 | Crizotinib | ROS1-positive NSCLC | 2018 | not available | 148,582 | 43,386 | 69.30% | non-hematological | yes | imported | no | oral | 2018 |
| 4 | Nilotinib | chronic myelogenous leukemia | 2018 | NCT00109707 | 99,030 | 32,120 | 76.00% | hematological | no | imported | no | oral | 2009 |
| 5 | Sintilimab | relapsed or refractory classical Hodgkin's lymphoma | 2019 | NCT03114683 | 27,231 | 9,877 | 80.40% | hematological | no | domestic | yes | intravenous | 2018 |
| 6 | Almonertinib | EGFR T790M advanced or metastatic NSCLC | 2020 | NCT02981108 | 52,401 | 18,822 | 68.90% | non-hematological | no | domestic | yes | oral | 2020 |
| 7 | Denosumab | giant cell tumor of bone | 2020 | NCT00680992 | 24,567 | 4,915 | 71.00% | non-hematological | yes | imported | yes | intravenous | 2019 |
| 8 | Camrelizumab | relapsed or refractory classical Hodgkin's lymphoma | 2020 | NCT03155425 | 77,467 | 11,456 | 77.30% | hematological | no | domestic | yes | intravenous | 2019 |
| 9 | Camrelizumab | advanced hepatocellular carcinoma | 2020 | NCT02989922 | 20,084 | 2,970 | 17.60% | non-hematological | no | domestic | yes | intravenous | 2020 |
| 10 | Toripalimab | advanced or metastatic Melanoma | 2020 | NCT03013101 | 10,433 | 2,871 | 17.30% | non-hematological | no | domestic | yes | intravenous | 2018 |
| 11 | Tislelizumab | relapsed or refractory classical Hodgkin's lymphoma | 2020 | NCT03209973 | 40,268 | 8,213 | 76.90% | hematological | no | domestic | yes | intravenous | 2019 |
| 12 | Tislelizumab | advanced or metastatic urothelial bladder cancer | 2020 | NCT04004221 | 15,488 | 3,159 | 24.80% | non-hematological | no | domestic | yes | intravenous | 2020 |
| 13 | Zanubrutinib | mantle cell lymphoma | 2020 | NCT03206970 | 84,738 | 47,513 | 83.70% | hematological | no | domestic | yes | oral | 2020 |
| 14 | Zanubrutinib | chronic lymphocytic leukemia/small lymphocytic lymphoma | 2020 | NCT03206918 | 42,983 | 24,101 | 84.60% | hematological | no | domestic | yes | oral | 2020 |
| 15 | Fluzoparib | ovarian cancer | 2021 | NCT03509636 | 28,369 | 16,145 | 69.90% | non-hematological | yes | domestic | yes | oral | 2020 |
| 16 | Orelabrutinib | mantle cell lymphoma | 2021 | NCT03494179 | 25,274 | 12,505 | 77.90% | hematological | no | domestic | yes | oral | 2020 |
| 17 | Orelabrutinib | chronic lymphocytic leukemia/ small lymphocytic lymphoma | 2021 | NCT03493217 | 25,107 | 12,422 | 73.80% | hematological | no | domestic | yes | oral | 2020 |
| 18 | Daratumumab | multiple myeloma | 2021 | NCT01985126 | 40,454 | 23,493 | 29.20% | hematological | no | imported | no | intravenous | 2019 |
| 19 | Ensartinib | advanced or metastatic NSCLC | 2021 | NCT03215693 | 60,302 | 17,811 | 51.90% | non-hematological | no | domestic | no | oral | 2020 |
| 20 | Furmonertinib | EGFR T790M advanced or metastatic NSCLC | 2021 | NCT03452592 | 51,569 | 10,649 | 74.10% | non-hematological | no | domestic | yes | oral | 2021 |
| 21 | Pamiparib | advanced high-grade ovarian cancer | 2021 | NCT03333915 | 26,865 | 12,342 | 68.30% | non-hematological | no | domestic | yes | oral | 2021 |
| 22 | Disitamab vedotin | advanced or metastatic gastric cancer | 2021 | not available | 56,520 | 15,909 | 24.40% | non-hematological | no | domestic | yes | intravenous | 2021 |
| 23 | Lorlatinib | NSCLC | 2022 | NCT01970865 | 45,532 | 29,323 | 35.60% | non-hematological | no | imported | no | oral | 2022 |
| 24 | Brentuximab | Hodgkin's lymphoma | 2022 | NCT00848926 | 41,734 | 19,242 | 53.89% | hematological | no | imported | no | intravenous | 2020 |
| 25 | Brentuximab | large-cell lymphoma | 2022 | NCT00866047 | 32,460 | 14,966 | 53.89% | hematological | no | imported | no | intravenous | 2020 |
| 26 | Savolitinib | NSCLC | 2022 | NCT02897479 | 37,696 | 23,220 | 38.40% | non-hematological | yes | domestic | yes | oral | 2021 |
| 27 | Carfilzomib | multiple myeloma | 2022 | NCT03029234 | 25,195 | 9,453 | 62.48% | non-hematological | no | imported | yes | intravenous | 2021 |
| Notes: NSCLC, non-small cell lung cancer. | | | | | | | | | | | | | |
