# Supplementary material for: Price negotiation and pricing of anticancer drugs in China: An observational study
Source: PLoS Med. 2024 Jan 2;21(1):e1004332. doi: 10.1371/journal.pmed.1004332 (PMC10793910; doi:10.1371/journal.pmed.1004332)
Supplement: S4 Table — (DOCX) [file pmed.1004332.s007.docx]

**S4 Table. Associations between treatment costs and clinical value, including control variables, for indications supported by randomized controlled trials before price negotiation in China**

| **Variables** | **Costs before negotiation** | | | | | | | | | | | | | | | | | | | |
| --- | --- | --- | --- | --- | --- | --- | --- | --- | --- | --- | --- | --- | --- | --- | --- | --- | --- | --- | --- | --- |
|  | **Model (1)** | | **Model (2)** | | **Model (3)** | | **Model (4)** | | **Model (5)** | | **Model (6)** | | **Model (7)** | | **Model (8)** | | **Model (9)** | | **Model (10)** | |
|  | **Coefficient (95% CI)** | ***P* value** | **Coefficient (95% CI)** | ***P* value** | **Coefficient (95% CI)** | ***P* value** | **Coefficient (95% CI)** | ***P* value** | **Coefficient (95% CI)** | ***P* value** | **Coefficient (95% CI)** | ***P* value** | **Coefficient (95% CI)** | ***P* value** | **Coefficient (95% CI)** | ***P* value** | **Coefficient (95% CI)** | ***P* value** | **Coefficient (95% CI)** | ***P* value** |
| Survival benefits in months | 0.034 (0.021, 0.048) | <0.001 | 0.032 (0.020, 0.044) | <0.001 | 0.032 (0.018, 0.046) | <0.001 | 0.033 (0.018, 0.048) | <0.001 | 0.034 (0.021, 0.048) | <0.001 | 0.032 (0.018, 0.046) | <0.001 | 0.034 (0.020, 0.047) | <0.001 | 0.035 (0.022, 0.048) | <0.001 | 0.034 (0.020, 0047) | <0.001 | 0.024 (0.008, 0.040) | 0.004 |
| QoL (ref = No difference) |  |  |  |  |  |  |  |  |  |  |  |  |  |  |  |  |  |  |  |  |
| Improvement | -0.070 (-0.28, 0.149) | 0.526 | -0.050 (-0.251, 0.150) | 0.618 | -0.064 (-0.283, 0.154) | 0.558 | -0.005 (-0.250, 0.240) | 0.965 | -0.070 (-0.291, 0.150) | 0.528 | -0.079 (-0.296, 0.139) | 0.473 | -0.087 (-0.312, 0.139) | 0.446 | -0.087 (-0.303, 0.130) | 0.428 | -0.093 (-0.321, 0.135) | 0.417 | -0.100 (-0.335, 0.134) | 0.395 |
| Reduction or unavailability | -0.145 (-0.349, 0.058) | 0.158 | -0.051 (-0.243, 0.141) | 0.596 | -0.126 (-0.331, 0.079) | 0.226 | -0.099 (-0.324, 0.127) | 0.386 | -0.145 (-0.350, 0.060) | 0.163 | -0.127 (-0.330, 0.075) | 0.214 | -0.168 (-0.383, 0.048) | 0.125 | -0.124 (-0.326, 0.078) | 0.223 | -0.171 (-0.385, 0.044) | 0.117 | -0.149 (-0.364, 0.067) | 0.173 |
| Safety (ref = Reduction) |  |  |  |  |  |  |  |  |  |  |  |  |  |  |  |  |  |  |  |  |
| Improvement or no difference | 0.134 (-0.089, 0.357) | 0.235 | 0.104 (-1.01, 0.309) | 0.314 | 0.118 (-0.106, 0.342) | 0.297 | 0.126 (-0.103, 0.356) | 0.276 | 0.135 (-0.091, 0.361) | 0.237 | 0.124 (-0.097, 0.346) | 0.267 | 0.111 (-0.123, 0.346) | 0.347 | 0.163 (-0.354, 0.026) | 0.149 | 0.121 (-0.106, 0.347) | 0.291 | 0.139 (-0.106, 0.384) | 0.261 |
| Domestically developed (ref = No) |  |  |  |  |  |  |  |  |  |  |  |  |  |  |  |  |  |  |  |  |
| Yes |  |  | -0.359 (-0.545, -0.173) | <0.001 |  |  |  |  |  |  |  |  |  |  |  |  |  |  |  |  |
| Year of approval (ref = Before 2017) |  |  |  |  |  |  |  |  |  |  |  |  |  |  |  |  |  |  |  |  |
| 2017 and beyond |  |  |  |  | 0.112 (-0.073, 0.297) | 0.232 |  |  |  |  |  |  |  |  |  |  |  |  |  |  |
| Cancer site (ref = Blood) |  |  |  |  |  |  |  |  |  |  |  |  |  |  |  |  |  |  |  |  |
| Lung |  |  |  |  |  |  | -0.151 (-0.468, 0.165) | 0.343 |  |  |  |  |  |  |  |  |  |  |  |  |
| Breast |  |  |  |  |  |  | -0.046 (-0.368, 0.276) | 0.775 |  |  |  |  |  |  |  |  |  |  |  |  |
| Colorectal |  |  |  |  |  |  | -0.134 (-0.552, 0.283) | 0.523 |  |  |  |  |  |  |  |  |  |  |  |  |
| Renal |  |  |  |  |  |  | 0.078 (0.350, 0.505) | 0.718 |  |  |  |  |  |  |  |  |  |  |  |  |
| Other |  |  |  |  |  |  | 0.024 (-0.240, 0.288) | 0.856 |  |  |  |  |  |  |  |  |  |  |  |  |
| First-line treatment (ref = No) |  |  |  |  |  |  |  |  |  |  |  |  |  |  |  |  |  |  |  |  |
| Yes |  |  |  |  |  |  |  |  | -0.009 (-0.185, 0.166) | 0.918 |  |  |  |  |  |  |  |  |  |  |
| Priority review (ref = No) |  |  |  |  |  |  |  |  |  |  |  |  |  |  |  |  |  |  |  |  |
| Yes |  |  |  |  |  |  |  |  |  |  | 0.135 (-0.040, 0.311) | 0.128 |  |  |  |  |  |  |  |  |
| Comparator (ref = Placebo) |  |  |  |  |  |  |  |  |  |  |  |  |  |  |  |  |  |  |  |  |
| Active |  |  |  |  |  |  |  |  |  |  |  |  | 0.066 (-0.137, 0.270) | 0.518 |  |  |  |  |  |  |
| Administration route (ref = Oral) |  |  |  |  |  |  |  |  |  |  |  |  |  |  |  |  |  |  |  |  |
| Intravenous |  |  |  |  |  |  |  |  |  |  |  |  |  |  | -0.164 (-0.353, 0.026) | 0.089 |  |  |  |  |
| Blind (ref = No) |  |  |  |  |  |  |  |  |  |  |  |  |  |  |  |  |  |  |  |  |
| Yes |  |  |  |  |  |  |  |  |  |  |  |  |  |  |  |  | -0.071 (-0.256, 0.114) | 0.447 |  |  |
| Baseline survival |  |  |  |  |  |  |  |  |  |  |  |  |  |  |  |  |  |  | 0.009 (0.002, 0.016) | 0.017 |
| Notes: We log-transformed treatment costs for these regression analyses. CI = confidence interval. QoL = quality of life. Of note, because associations were strongly influenced by the outlier(s), the Rituximab for the treatment of diffuse large-B-cell lymphoma, we excluded the outlier(s) from these analyses. | | | | | | | | | | | | | | | | | | | | |
