# Supplementary material for: Price negotiation and pricing of anticancer drugs in China: An observational study
Source: PLoS Med. 2024 Jan 2;21(1):e1004332. doi: 10.1371/journal.pmed.1004332 (PMC10793910; doi:10.1371/journal.pmed.1004332)
Supplement: S5 Table — (DOCX) [file pmed.1004332.s008.docx]

**S5 Table. Associations between treatment costs and clinical value, including control variables, for indications supported by randomized controlled trials after price negotiation in China**

| **Variables** | **Costs after negotiation** | | | | | | | | | | | | | | | | | | | |
| --- | --- | --- | --- | --- | --- | --- | --- | --- | --- | --- | --- | --- | --- | --- | --- | --- | --- | --- | --- | --- |
|  | **Model (1)** | | **Model (2)** | | **Model (3)** | | **Model (4)** | | **Model (5)** | | **Model (6)** | | **Model (7)** | | **Model (8)** | | **Model (9)** | | **Model (10)** | |
|  | **Coefficient (95% CI)** | ***P* value** | **Coefficient (95% CI)** | ***P* value** | **Coefficient (95% CI)** | ***P* value** | **Coefficient (95% CI)** | ***P* value** | **Coefficient (95% CI)** | ***P* value** | **Coefficient (95% CI)** | ***P* value** | **Coefficient (95% CI)** | ***P* value** | **Coefficient (95% CI)** | ***P* value** | **Coefficient (95% CI)** | ***P* value** | **Coefficient (95% CI)** | ***P* value** |
| Survival benefits in months | 0.032 (0.019, 0.044) | <0.001 | 0.029 (0.018, 0.040) | <0.001 | 0.031 (0.018, 0.044) | <0.001 | 0.028 (0.014, 0,042) | <0.001 | 0.031 (0.019, 0.044) | <0.001 | 0.031 (0.018, 0.044) | <0.001 | 0.031 (0.019, 0.044) | <0.001 | 0.032 (0.020, 0.044) | <0.001 | 0.031 (0.019, 0.044) | <0.001 | 0.024 (0.009, 0.039) | 0.002 |
| QoL (ref = No difference) |  |  |  |  |  |  |  |  |  |  |  |  |  |  |  |  |  |  |  |  |
| Improvement | -0.102 (-0.305, 0.100) | 0.317 | -0.081 (-0.258, 0.096) | 0.365 | -0.102 (-0.306. 0.102) | 0.324 | -0.019 (-0.242, 0.205) | 0.868 | -0.103 (-0.307, 0.102) | 0.319 | -0.105 (-0.309, 0.099) | 0.309 | -0.113 (-0.323, 0.096) | 0.285 | -0.116 (-0.318, 0.087) | 0.258 | -0.113 (-0.324, 0.099) | 0.293 | -0.122 (-0.341, 0.097) | 0.271 |
| Reduction or unavailability | -0.167 (-0.355, 0.022) | 0.082 | -0.063 (-0.233, 0.107) | 0.463 | -0.264 (-0.356, 0.027) | 0.092 | -0.102 (-0.143, 0.276) | 0.327 | -0.166 (-0.356, 0.024) | 0.086 | -0.162 (-0.352, 0.029) | 0.095 | -0,181 (-0.380, 0.019) | 0.075 | -0.150 (-0.339, 0.038) | 0.116 | -0.178 (-0.377, 0.022) | 0.080 | -0.151 (-0.352, 0.050) | 0.138 |
| Safety (ref = Reduction) |  |  |  |  |  |  |  |  |  |  |  |  |  |  |  |  |  |  |  |  |
| Improvement or no difference | 0.083 (-0.124, 0.289) | 0.428 | 0.049 (-0.131, 0.230) | 0.587 | 0.081 (-0.129, 0.290) | 0.446 | 0.066 (-0.143, 0.276) | 0.531 | 0.084 (-0.125, 0.293) | 0.425 | 0.080 (-0.128, 0.288) | 0.447 | 0.068 (-0.149, 0.286) | 0.533 | -0.127 (-0.304, 0.050) | 0.316 | -0.077 (-0.134, 0.287) | 0.469 | 0.068 (-0.161, 0.297) | 0.556 |
| Domestically developed (ref = No) |  |  |  |  |  |  |  |  |  |  |  |  |  |  |  |  |  |  |  |  |
| Yes |  |  | -0.396 (-0.561, -0.232) | <0.001 |  |  |  |  |  |  |  |  |  |  |  |  |  |  |  |  |
| Year of approval (ref = Before 2017) |  |  |  |  |  |  |  |  |  |  |  |  |  |  |  |  |  |  |  |  |
| 2017 and beyond |  |  |  |  | 0.014 (-0.159, 0.187) | 0.873 |  |  |  |  |  |  |  |  |  |  |  |  |  |  |
| Cancer site (ref = Blood) |  |  |  |  |  |  |  |  |  |  |  |  |  |  |  |  |  |  |  |  |
| Lung |  |  |  |  |  |  | -0.258 (-0.546, 0.031) | 0.079 |  |  |  |  |  |  |  |  |  |  |  |  |
| Breast |  |  |  |  |  |  | -0.190 (-0.484, 0.103) | 0.200 |  |  |  |  |  |  |  |  |  |  |  |  |
| Colorectal |  |  |  |  |  |  | -0.215 (-0.596, 0.165) | 0.263 |  |  |  |  |  |  |  |  |  |  |  |  |
| Renal |  |  |  |  |  |  | -0.011 (-0.401, 0.379) | 0.957 |  |  |  |  |  |  |  |  |  |  |  |  |
| Other |  |  |  |  |  |  | -0.075 (-0.315, 0.166) | 0.539 |  |  |  |  |  |  |  |  |  |  |  |  |
| First-line treatment (ref = No) |  |  |  |  |  |  |  |  |  |  |  |  |  |  |  |  |  |  |  |  |
| Yes |  |  |  |  |  |  |  |  | -0.012 (-0.175, 0.150) | 0.881 |  |  |  |  |  |  |  |  |  |  |
| Priority review (ref = No) |  |  |  |  |  |  |  |  |  |  |  |  |  |  |  |  |  |  |  |  |
| Yes |  |  |  |  |  |  |  |  |  |  | 0.037 (-0.128, 0.202) | 0.655 |  |  |  |  |  |  |  |  |
| Comparator (ref = Placebo) |  |  |  |  |  |  |  |  |  |  |  |  |  |  |  |  |  |  |  |  |
| Active |  |  |  |  |  |  |  |  |  |  |  |  | 0.042 (-0.147, 0.231) | 0.659 |  |  |  |  |  |  |
| Administration route (ref = Oral) |  |  |  |  |  |  |  |  |  |  |  |  |  |  |  |  |  |  |  |  |
| Intravenous |  |  |  |  |  |  |  |  |  |  |  |  |  |  | -0.127 (-0.304, 0.050) | 0.155 |  |  |  |  |
| Blind (ref = No) |  |  |  |  |  |  |  |  |  |  |  |  |  |  |  |  |  |  |  |  |
| Yes |  |  |  |  |  |  |  |  |  |  |  |  |  |  |  |  | -0.031 (-0.203, 0.141) | 0.723 |  |  |
| Baseline survival |  |  |  |  |  |  |  |  |  |  |  |  |  |  |  |  |  |  | 0.006 (-0.000, 0.013) | 0.068 |
| Notes: We log-transformed treatment costs for these regression analyses. CI = confidence interval. QoL = quality of life. Of note, because associations were strongly influenced by the outlier(s), the Rituximab for the treatment of diffuse large-B-cell lymphoma, we excluded the outlier(s) from these analyses. | | | | | | | | | | | | | | | | | | | | |
