# Supplementary material for: Price negotiation and pricing of anticancer drugs in China: An observational study
Source: PLoS Med. 2024 Jan 2;21(1):e1004332. doi: 10.1371/journal.pmed.1004332 (PMC10793910; doi:10.1371/journal.pmed.1004332)
Supplement: S6 Table — (DOCX) [file pmed.1004332.s009.docx]

**S6 Table. Associations between treatment costs and clinical value, including interaction terms and control variables, for indications supported by randomized controlled trials in China.**

| **Variables** | **Costs before and after negotiation** | | | | | | | | | | | | | | | | | | | |
| --- | --- | --- | --- | --- | --- | --- | --- | --- | --- | --- | --- | --- | --- | --- | --- | --- | --- | --- | --- | --- |
|  | **Model (1)** | | **Model (2)** | | **Model (3)** | | **Model (4)** | | **Model (5)** | | **Model (6)** | | **Model (7)** | | **Model (8)** | | **Model (9)** | | **Model (10)** | |
|  | **Coefficient (95% CI)** | ***P* value** | **Coefficient (95% CI)** | ***P* value** | **Coefficient (95% CI)** | ***P* value** | **Coefficient (95% CI)** | ***P* value** | **Coefficient (95% CI)** | ***P* value** | **Coefficient (95% CI)** | ***P* value** | **Coefficient (95% CI)** | ***P* value** | **Coefficient (95% CI)** | ***P* value** | **Coefficient (95% CI)** | ***P* value** | **Coefficient (95% CI)** | ***P* value** |
| Survival benefits in months | 0.034 (0.022, 0.047) | <0.001 | 0.032 (0.021, 0.043) | <0.001 | 0.033 (0.020, 0.046) | <0.001 | 0.032 (0.018, 0.045) | <0.001 | 0.034 (0.022, 0.047) | <0.001 | 0.033 (0.020, 0.046) | <0.001 | 0.034 (0.021, 0.047) | <0.001 | 0.035 (0.022, 0.048) | <0.001 | 0.034 (0.021, 0.047) | <0.001 | 0.029 (0.016, 0.042) | <0.001 |
| QoL (ref = No difference) |  |  |  |  |  |  |  |  |  |  |  |  |  |  |  |  |  |  |  |  |
| Improvement | -0.070 (-0.279, 0.139) | 0.510 | -0.049 (-0.236, 0.137) | 0.602 | -0.067 (-0.276, 0.142) | 0.529 | 0.004 (-0.214, 0.223) | 0.969 | -0.070 (-0.280, 0.140) | 0.510 | -0.075 (-0.284, 0.133) | 0.476 | -0.084 (-0.296, 0.129) | 0.438 | -0.085 (-0.291, 0.122) | 0.419 | -0.087 (-0.300, 0.127) | 0.424 | -0.081 (-0.284, 0.121) | 0.427 |
| Reduction or unavailability | -0.145 (-0.340, 0.049) | 0.141 | -0.046 (-0.222, 0.130) | 0.603 | -0.134 (-0.330, 0.061) | 0.176 | -0.090 (-0.291, 0.112) | 0.381 | -0.144 (-0.340, 0.050) | 0.144 | -0.134 (-0.328, 0.060) | 0.174 | -0.163 (-0.363, 0.036) | 0.108 | -0.127 (-0.319, 0.065) | 0.194 | -0.164 (-0.363, 0.036) | 0.107 | -0.153 (-0.341, 0.035) | 0.109 |
| Safety (ref = Reduction) |  |  |  |  |  |  |  |  |  |  |  |  |  |  |  |  |  |  |  |  |
| Improvement or no difference | 0.134 (-0.079, 0.347) | 0.216 | 0.102 (-0.088, 0.293) | 0.290 | 0.125 (-0.089, 0.339) | 0.250 | 0.122 (-0.091, 0.335) | 0.260 | 0.135 (-0.079, 0.350) | 0.215 | 0.128 (-0.085, 0.340) | 0.237 | 0.116 (-0.103, 0.334) | 0.298 | 0.160 (-0.052, 0.371) | 0.138 | 0.124 (-0.090, 0.339) | 0.254 | 0.154 (-0.053, 0.361) | 0.143 |
| Negotiation (ref = Before negotiation) |  |  |  |  |  |  |  |  |  |  |  |  |  |  |  |  |  |  |  |  |
| After negotiation | -0.369 (-0.602, -0.137) | 0.002 | -0.369 (-0.577, -0.162) | <0.001 | -0.369 (-0.602, -0.137) | 0.002 | -0.369 (-0.600, -0.138) | 0.002 | -0.369 (-0.603, -0.136) | 0.002 | -0.369 (-0.601, -0.138) | 0.002 | -0.369 (-0.602, -0.137) | 0.002 | -0.369 (-0.599, -0.140) | 0.002 | -0.369 (-0.602, -0.137) | 0.002 | -0.369 (-0.594, -0.144) | 0.001 |
| Negotiation × Survival benefits (ref=Before negotiation × Survival benefits) |  |  |  |  |  |  |  |  |  |  |  |  |  |  |  |  |  |  |  |  |
| After negotiation × Survival benefits | -0.003 (-0.021, 0.015) | 0.751 | -0.003 (-0.500, -0.255) | 0.722 | -0.003 (-0.021, 0.015) | 0.751 | -0.003 (-0.021, 0.015) | 0.750 | -0.003 (-0.021, 0.015) | 0.752 | -0.003 (-0.021, 0.015) | 0.750 | -0.003 (-0.021, 0.015) | 0.752 | -0.003 (-0.021, 0.015) | 0.748 | -0.003 (-0.021, 0.015) | 0.751 | -0.003 (-0.020, 0.015) | 0.743 |
| Negotiation × QoL (ref = Before negotiation × No difference) |  |  |  |  |  |  |  |  |  |  |  |  |  |  |  |  |  |  |  |  |
| After negotiation × Improvement | -0.033 (-0.328, 0.263) | 0.828 | -0.033 (-0.296, 0.231) | 0.807 | -0.033 (-0.328,0.263) | 0.828 | -0.033 (-0.326, 0.261) | 0.827 | -0.033 (-0.329, 0.264) | 0.828 | -0.033 (-0.327, 0.262) | 0.827 | -0.033 (-0.329, 0.264) | 0.828 | -0.033 (-0.324, 0.259) | 0.825 | -0.033 (-0329, 0.263) | 0.828 | -0.033 (-0.319, 0.253) | 0.822 |
| After negotiation × Reduction or unavailability | -0.021 (-0.296, 0.254) | 0.879 | -0.021 (-0.266, 0.224) | 0.864 | -0.021 (-0.296, 0.254) | 0.879 | -0.021 (-0.294, 0.252) | 0.879 | -0.021 (-0.297, 0.254) | 0.879 | -0.021 (-0.294, 0.253) | 0.879 | -0.021 (-0.296, 0.254) | 0.879 | -0.021 (0.292,0.250) | 0.878 | -0.021 (-0.296, 0.254) | 0.879 | -0.021 (-0.287, 0.245) | 0.875 |
| Negotiation × Safety (ref = Before negotiation × Reduction) |  |  |  |  |  |  |  |  |  |  |  |  |  |  |  |  |  |  |  |  |
| After negotiation × Improvement or no difference | -0.051 (-0.353, 0.250) | 0.737 | -0.051 (-0.320, 0.218) | 0.706 | -0.051 (-0.353, 0.250) | 0.736 | -0.051 (-0.351, 0.248) | 0.735 | -0.051 (-0.353, 0.251) | 0.738 | -0.051 (-0.352, 0.249) | 0.736 | -0.051 (-0.353, 0.251) | 0.727 | -0.051 (-0.349, 0.246) | 0.733 | -0.051 (-0.353, 0.251) | 0.737 | -0.051 (-0.343, 0.240) | 0.728 |
| Domestically developed (ref = No) |  |  |  |  |  |  |  |  |  |  |  |  |  |  |  |  |  |  |  |  |
| Yes |  |  | -0.378 (-0.500, -0.255) | <0.001 |  |  |  |  |  |  |  |  |  |  |  |  |  |  |  |  |
| Year of approval (ref = Before 2017) |  |  |  |  |  |  |  |  |  |  |  |  |  |  |  |  |  |  |  |  |
| 2017 and beyond |  |  |  |  | 0.063 (-0.353, 0.250) | 0.323 |  |  |  |  |  |  |  |  |  |  |  |  |  |  |
| Cancer site (ref = Blood) |  |  |  |  |  |  |  |  |  |  |  |  |  |  |  |  |  |  |  |  |
| Lung |  |  |  |  |  |  | -0.204 (-0.413, 0.004) | 0.054 |  |  |  |  |  |  |  |  |  |  |  |  |
| Breast |  |  |  |  |  |  | -0.118 (-0.330, 0.094) | 0.272 |  |  |  |  |  |  |  |  |  |  |  |  |
| Colorectal |  |  |  |  |  |  | -0.174 (-0.450, 0.100) | 0.211 |  |  |  |  |  |  |  |  |  |  |  |  |
| Renal |  |  |  |  |  |  | 0.033 (-0.248, 0.315) | 0.814 |  |  |  |  |  |  |  |  |  |  |  |  |
| Other |  |  |  |  |  |  | -0.025 (-0.199, 0.148) | 0.774 |  |  |  |  |  |  |  |  |  |  |  |  |
| First-line treatment (ref = No) |  |  |  |  |  |  |  |  |  |  |  |  |  |  |  |  |  |  |  |  |
| Yes |  |  |  |  |  |  |  |  | -0.011 (-0.129, 0.107) | 0.859 |  |  |  |  |  |  |  |  |  |  |
| Priority review (ref = No) |  |  |  |  |  |  |  |  |  |  |  |  |  |  |  |  |  |  |  |  |
| Yes |  |  |  |  |  |  |  |  |  |  | 0.086 (-0.033, 0.205) | 0.154 |  |  |  |  |  |  |  |  |
| Comparator (ref = Placebo) |  |  |  |  |  |  |  |  |  |  |  |  |  |  |  |  |  |  |  |  |
| Active |  |  |  |  |  |  |  |  |  |  |  |  | 0.054 (-0.083, 0.191) | 0.436 |  |  |  |  |  |  |
| Administration route (ref = Oral) |  |  |  |  |  |  |  |  |  |  |  |  |  |  |  |  |  |  |  |  |
| Intravenous |  |  |  |  |  |  |  |  |  |  |  |  |  |  | -0.146 (-0.274, -0.018) | 0.026 |  |  |  |  |
| Blind (ref = No) |  |  |  |  |  |  |  |  |  |  |  |  |  |  |  |  |  |  |  |  |
| Yes |  |  |  |  |  |  |  |  |  |  |  |  |  |  |  |  | -0.051 (-0.176, 0.074) | 0.422 |  |  |
| Baseline survival |  |  |  |  |  |  |  |  |  |  |  |  |  |  |  |  |  |  | 0.008 (0.003, 0.012) | 0.001 |
| Notes: We log-transformed treatment costs for these regression analyses. CI = confidence interval. QoL = quality of life. Negotiation × Clinical value (survival benefits, QoL, or safety) refers to the interaction term involving negotiation and measures of clinical value. Of note, because associations were strongly influenced by the outlier(s), the Rituximab for the treatment of diffuse large-B-cell lymphoma, we excluded the outlier(s) from these analyses. | | | | | | | | | | | | | | | | | | | | |
