# Supplementary material for: Price negotiation and pricing of anticancer drugs in China: An observational study
Source: PLoS Med. 2024 Jan 2;21(1):e1004332. doi: 10.1371/journal.pmed.1004332 (PMC10793910; doi:10.1371/journal.pmed.1004332)
Supplement: S7 Table — (DOCX) [file pmed.1004332.s010.docx]

**S7 Table. Associations between treatment costs and ORR, including control variables, for indications supported by single-arm clinical trials before price negotiation in China.**

| **Variables** | **Costs before negotiation** | | | | | | | | | | | | | |
| --- | --- | --- | --- | --- | --- | --- | --- | --- | --- | --- | --- | --- | --- | --- |
|  | **Model (1)** | | **Model (2)** | | **Model (3)** | | **Model (4)** | | **Model (5)** | | **Model (6)** | | **Model (7)** | |
|  | **Coefficient (95% CI)** | ***P* value** | **Coefficient (95% CI)** | ***P* value** | **Coefficient (95% CI)** | ***P* value** | **Coefficient (95% CI)** | ***P* value** | **Coefficient (95% CI)** | ***P* value** | **Coefficient (95% CI)** | ***P* value** | **Coefficient (95% CI)** | ***P* value** |
| ORR | 0.597 (0.163, 1.030) | 0.009 | 0.687 (0.221, 1.153) | 0.006 | 0.586 (0.143, 1.028) | 0.012 | 0.540 (0.077, 1.002) | 0.024 | 0.574 (0.171, 0.977) | 0.007 | 0.588 (0156, 1.019) | 0.010 | 0.608 (0.169, 1.046) | 0.009 |
| Cancer site (ref = hematological) |  |  |  |  |  |  |  |  |  |  |  |  |  |  |
| Non-hematological |  |  | 0.110 (-0.100, 0.321) | 0.290 |  |  |  |  |  |  |  |  |  |  |
| First-line treatment (ref = No) |  |  |  |  |  |  |  |  |  |  |  |  |  |  |
| Yes |  |  |  |  | 0.079 (-0.202, 0.361) | 0.566 |  |  |  |  |  |  |  |  |
| Administration route (ref = Oral) |  |  |  |  |  |  |  |  |  |  |  |  |  |  |
| Intravenous |  |  |  |  |  |  | -0.080 (-0.289, 0.128) | 0.436 |  |  |  |  |  |  |
| Conditional approval (ref = No) |  |  |  |  |  |  |  |  |  |  |  |  |  |  |
| Yes |  |  |  |  |  |  |  |  | -0.219 (-0.418, -0.019) | 0.033 |  |  |  |  |
| Domestically developed (ref = No) |  |  |  |  |  |  |  |  |  |  |  |  |  |  |
| Yes |  |  |  |  |  |  |  |  |  |  | -0.118 (-0.325, 0.088) | 0.249 |  |  |
| Year of approval (ref = Before 2017) |  |  |  |  |  |  |  |  |  |  |  |  |  |  |
| 2017 and beyond |  |  |  |  |  |  |  |  |  |  |  |  | -0.110 (-0.389, 0.169) | 0.424 |
| Notes: We log-transformed treatment costs for these regression analyses. CI = confidence interval. ORR: overall response rate. | | | | | | | | | | | | | | |
