# Supplementary material for: Price negotiation and pricing of anticancer drugs in China: An observational study
Source: PLoS Med. 2024 Jan 2;21(1):e1004332. doi: 10.1371/journal.pmed.1004332 (PMC10793910; doi:10.1371/journal.pmed.1004332)
Supplement: S8 Table — (DOCX) [file pmed.1004332.s011.docx]

**S8 Table. Associations between treatment costs and ORR, including control variables, for indications supported by single-arm clinical trials after price negotiation in China.**

| **Variables** | **Costs after negotiation** | | | | | | | | | | | | | |
| --- | --- | --- | --- | --- | --- | --- | --- | --- | --- | --- | --- | --- | --- | --- |
|  | **Model (1)** | | **Model (2)** | | **Model (3)** | | **Model (4)** | | **Model (5)** | | **Model (6)** | | **Model (7)** | |
|  | **Coefficient (95% CI)** | ***P* value** | **Coefficient (95% CI)** | ***P* value** | **Coefficient (95% CI)** | ***P* value** | **Coefficient (95% CI)** | ***P* value** | **Coefficient (95% CI)** | ***P* value** | **Coefficient (95% CI)** | ***P* value** | **Coefficient (95% CI)** | ***P* value** |
| ORR | 0.688 (0.179, 1.197) | 0.010 | 0.684 (0.124, 1.244) | 0.019 | 0.675 (0.156, 1.194) | 0.013 | 0.496 (0.007, 0.985) | 0.047 | 0.656 (0.207, 1.105) | 0.006 | 0.674 (0.178, 1.171) | 0.010 | 0.707 (-0.507, 0.138) | 0.008 |
| Cancer site (ref = hematological) |  |  |  |  |  |  |  |  |  |  |  |  |  |  |
| Non-hematological |  |  | -0.005 (-0.258, 0.248) | 0.966 |  |  |  |  |  |  |  |  |  |  |
| First-line treatment (ref = No) |  |  |  |  |  |  |  |  |  |  |  |  |  |  |
| Yes |  |  |  |  | 0.096 (-0.234, 0.426) | 0.552 |  |  |  |  |  |  |  |  |
| Administration route (ref = Oral) |  |  |  |  |  |  |  |  |  |  |  |  |  |  |
| Intravenous |  |  |  |  |  |  | -0.270 (-0.491, -0.049) | 0.019 |  |  |  |  |  |  |
| Conditional approval (ref = No) |  |  |  |  |  |  |  |  |  |  |  |  |  |  |
| Yes |  |  |  |  |  |  |  |  | -0.311 (-0.533, -0.089) | 0.008 |  |  |  |  |
| Domestically developed (ref = No) |  |  |  |  |  |  |  |  |  |  |  |  |  |  |
| Yes |  |  |  |  |  |  |  |  |  |  | -0.180 (-0.418, 0.058) | 0.132 |  |  |
| Year of approval (ref = Before 2017) |  |  |  |  |  |  |  |  |  |  |  |  |  |  |
| 2017 and beyond |  |  |  |  |  |  |  |  |  |  |  |  | -0.185 (-0.507, 0.138) | 0.249 |
| Notes: We log-transformed treatment costs for these regression analyses. CI = confidence interval. ORR: overall response rate. | | | | | | | | | | | | | | |
