# Supplementary material for: Price negotiation and pricing of anticancer drugs in China: An observational study
Source: PLoS Med. 2024 Jan 2;21(1):e1004332. doi: 10.1371/journal.pmed.1004332 (PMC10793910; doi:10.1371/journal.pmed.1004332)
Supplement: S9 Table — (DOCX) [file pmed.1004332.s012.docx]

**S9 Table. Associations between treatment costs and ORR, including interaction terms and control variables, for indications supported by single-arm clinical trials in China.**

| **Variables** | **Costs before and after negotiation** | | | | | | | | | | | | | |
| --- | --- | --- | --- | --- | --- | --- | --- | --- | --- | --- | --- | --- | --- | --- |
|  | **Model (1)** | | **Model (2)** | | **Model (3)** | | **Model (4)** | | **Model (5)** | | **Model (6)** | | **Model (7)** | |
|  | **Coefficient (95% CI)** | ***P* value** | **Coefficient (95% CI)** | ***P* value** | **Coefficient (95% CI)** | ***P* value** | **Coefficient (95% CI)** | ***P* value** | **Coefficient (95% CI)** | ***P* value** | **Coefficient (95% CI)** | ***P* value** | **Coefficient (95% CI)** | ***P* value** |
| ORR | 0.596 (0.140, 1.051) | 0.011 | 0.636 (0.159, 1.112) | 0.010 | 0.584 (0.126, 1.042) | 0.014 | 0.479 (0.028, 0.930) | 0.038 | 0.569 (0.160, 0.978) | 0.007 | 0.584 (0.141, 1.027) | 0.011 | 0.584 (0.126, 1.042) | 0.014 |
| Negotiation (ref = Before negotiation) |  |  |  |  |  |  |  |  |  |  |  |  |  |  |
| After negotiation | -0.495 (-0.899, -0.090) | 0.018 | -0.485 (-0.901, -0.088) | 0.018 | -0.495 (-0.900, -0.089) | 0.018 | -0.495 (-0.884, -0.105) | 0.014 | -0.495 (-0.857, -0.132) | 0.008 | -0.495 (-0.887, -0.102) | 0.015 | -0.495 (-0.900, -0.089) | 0.018 |
| Negotiation × ORR (ref = Before negotiation × ORR) |  |  |  |  |  |  |  |  |  |  |  |  |  |  |
| ORR × After negotiation | 0.092 (-0.552, 0.735) | 0.776 | 0.092 (-0.557, 0.740) | 0.778 | 0.092 (-0.555, 0.738) | 0.777 | 0.091 (-0.529, 0.712) | 0.768 | 0.092 (-0.486, 0.669) | 0.751 | 0.092 (-0.535, 0.718) | 0.770 | 0.092 (-0.555, 0.738) | 0.777 |
| Cancer site (ref = hematological) |  |  |  |  |  |  |  |  |  |  |  |  |  |  |
| Non-hematological |  |  | 0.049 (-0.109, 0.206) | 0.538 |  |  |  |  |  |  |  |  |  |  |
| First-line treatment (ref = No) |  |  |  |  |  |  |  |  |  |  |  |  |  |  |
| Yes |  |  |  |  | 0.086 (-0.121, 0.292) | 0.408 |  |  |  |  |  |  |  |  |
| Administration route (ref = Oral) |  |  |  |  |  |  |  |  |  |  |  |  |  |  |
| Intravenous |  |  |  |  |  |  | -0.164 (-0.313, -0.016) | 0.030 |  |  |  |  |  |  |
| Conditional approval (ref = No) |  |  |  |  |  |  |  |  |  |  |  |  |  |  |
| Yes |  |  |  |  |  |  |  |  | -0.259 (-0.402, -0.116) | 0.001 |  |  |  |  |
| Domestically developed (ref = No) |  |  |  |  |  |  |  |  |  |  |  |  |  |  |
| Yes |  |  |  |  |  |  |  |  |  |  | -0.149 (-0.299, 0.002) | 0.052 |  |  |
| Year of approval (ref = Before 2017) |  |  |  |  |  |  |  |  |  |  |  |  |  |  |
| 2017 and beyond |  |  |  |  |  |  |  |  |  |  |  |  | 0.086 (-0.121, 0.292) | 0.408 |
| Notes: We log-transformed treatment costs for these regression analyses. CI = confidence interval. ORR: overall response rate. Negotiation × ORR refers to the interaction term involving negotiation and ORR. | | | | | | | | | | | | | | |
