# Supplementary material for: Price negotiation and pricing of anticancer drugs in China: An observational study
Source: PLoS Med. 2024 Jan 2;21(1):e1004332. doi: 10.1371/journal.pmed.1004332 (PMC10793910; doi:10.1371/journal.pmed.1004332)
Supplement: Attachment — Submitted filename: 231011-Rebuttal letter.pdf [file pmed.1004332.s013.pdf]

Reply to Reviewer 1's Comments for "Impact of price negotiation on the pricing of anticancer drugs in China" (PMEDICINE-D-23-01449R1)

We thank the reviewers for providing all the valuable and helpful comments on our manuscript. We have made substantial efforts to revise our paper based on these comments. The associated changes are highlighted in red color in the revised manuscript. The following are responses to the comments in order of occurrence.

> **1. The English of your manuscript needs to be improved before resubmission, especially paying attention to initial size (e.g. P4, the first letter of the "indications" in the seventh to last line needs to be capitalized) and linguistic logic (e.g. P8,"Re-estimating regression models for indication supported by double-arm clinical trials with OS data as the only survival measure, with PFS data as the only survival measure, or without anticancer drugs launched before 2017 yielded highly similar results." Three scenarios were classified to evaluate the robustness, but the language expression was somewhat unclear and needed to be modified).**

We appreciate the referee's careful reading. Following this comment, we have gone through the whole manuscript for many times to improve the language expressions.

> **2. The unit of measurement in this manuscript was suggested to be unified into US dollars, because you quoted the data in USD in the background and CNY in the results.**

We have accordingly revised the manuscript to use US dollars as the unit of measurement in the study. We converted treatment costs to US dollars by applying the exchange rates for the respective years of negotiation, obtaining from the OECD.Stat: Organisation for Economic Co-operation and Development's statistical database (*lines 167-170, page 6 in revised-track version*).

> **3. As P5 showed that the expected treatment duration was obtained from drug label, generally speaking, the time of each drug was different, such as drug A should take orally every day with a cycle of 28 days; drug B was injected every 3 weeks, is the expected treatment duration calculated as a cycle? In other words, could you explain in detail the estimation of the expected treatment duration?**

> **4. "The median treatment duration of each indication was collected from the pivotal trial"， is it just to extract the median follow-up time of key indicators such as safety, PFS/OS, ORR, etc.?**

Thank the referee for giving us the opportunity to clarify how we estimated the treatment duration. Since the two questions are highly related, we merged them together. The expected treatment durations for therapeutic indications were median treatment durations extracted from corresponding pivotal clinical trials. It's important to note that the median treatment duration in our study did not refer to the median follow-up time of key indicators such as safety, PFS/OS, ORR, etc. Typically, pivotal clinical trial publications report median treatment durations in the 'safety' section, expressed as median numbers of cycles, median durations of exposure, median treatment times, median numbers of infusions, and so on. The treatment costs over the expected treatment durations were calculated based on prices and dosing information from drug labels. While the expected treatment durations were median treatment durations extracted from corresponding pivotal clinical trials, the dosing information was extracted from drug labels. We have revised the expression in the manuscript to make it more straightforward as "the median treatment duration for each indication was collected from the pivotal trial, representing the expected treatment duration when calculating treatment costs" (*lines 164, page 6 in revised-track version*).

5. In the Methods section, it was mentioned that when there were multiple clinical trials, these involving Chinese or Asian population were preferred, but would the relevant results also be included when trials only based in Europe or other regions (excluding Chinese and Asian population)? The indicators extracted in this way were biased and may not represent the clinical value of the drug in the Chinese population.

Thank the referee for the insightful comment. We would include the pivotal clinical trials based on Europe or other regions for several reasons.

First, all the clinical trials examined in our study were pivotal clinical trials that supported the approval of the therapeutic indications included in our study. These trials had to meet the requirements set forth by and be recognized by the National Medical Products Administration (NMPA).

Second, the NMPA places emphasis on considering racial factors and conducting benefit/risk evaluations in Chinese patients. For drugs with no relevant data on the Chinese population in the global dataset, but with sufficient racial factor-related study and analysis data where no significant impact of racial factors is identified, marketing approval can be considered on the premise of strict risk control. Further details can be found in the official document "Clinical Technical Requirements for Drugs Marketed Overseas but Not Marketed in China" (http://english.nmpa.gov.cn/2020-11/18/c_568155.htm) updated in 2020 in English version.

Third, the National Healthcare Security Administration (NHSA) emphasizes strengthening the connection between price negotiation and the review and approval processes, with the aim of facilitating

patient access and meeting their clinical needs. Therefore, it is likely that the NHSA reviewed the same efficacy and safety profiles at the time of price negotiation.

In this context, we would include pivotal clinical trials conducted in Europe or other regions (excluding Chinese and Asian populations) because they were recognized by the NMPA and aligned with regulatory and price negotiation policies. While acknowledging that the clinical value generated from these trials may have some bias, these trials could provide valuable supporting information for price negotiation at the time.

The following is a brief introduction of China's regulatory reform in recent years concerning the clinical trial approval system in case the referee is interested:

China has faced significant delays in the availability and timing of new drugs for an extended period, primarily attributable to regulatory issues such as severe application backlogs, lengthy regulatory review times, and the clinical trial approval system (Li and Yang, 2021; Luo et al., 2021). To address challenges within the clinical trial approval system, measures were initiated in 2015, and since 2018, clinical trial data from outside the country has been accepted for Chinese approval (Table S1) (Luo et al., 2021).

Table S1 Drug regulatory reform related to clinical trials in reducing drug lags in China.

**Initiation date**	**Drug regulatory reforms**	**Department**	**Measures aimed at reducing delays related to clinical trials**	**Target drugs**
2015-08-18	Opinions on reforming the review and approval system for drugs and medical devices	State Council	Allow unmarketed new drugs outside China to conduct clinical trials simultaneously in China	Drugs for the treatment of acquired immune deficiency syndrome; malignant tumors; infectious diseases; rare diseases and etc.
2017-10-08	Opinions on deepening the reform of the drug review and approval system and encouraging the innovation of drugs and medical devices	General Office of the State Council	• Allow for conditional approval based on data from Phase 1 and Phase 2 clinical trials;<br>• Allow the use of data from multi-regional clinical trials (MRCT);	All drugs
2018-07-06	Guidelines for acceptance of data from overseas clinical trials of pharmaceutical products	NMPA	Acceptance of clinical trial data from outside China to expedite new drug launches	All drugs

References

Li X, Yang Y. The drug lag issue: a 20-year review of China. Investigational new drugs 2021; 39(5): 1389-98.

Luo X, Du X, Li Z, Qian F, Yang Y. Assessment of the Delay in Novel Anticancer Drugs between China and the United States: A Comparative Study of Drugs Approved between 2010 and 2021. Clin Pharmacol Ther 2023; 113(1): 170-81.

6. The discussion part is about the expansion and analysis of the results, and there is no need for the expression shown in Table 1 and Figure 1.

We thank the referee for the suggestion. We have accordingly deleted such expression.

We wish the referee would be satisfied with our revision.

Reply to Reviewer 2's Comments for "Impact of price negotiation on the pricing of anticancer drugs in China" (PMEDICINE-D-23-01449R1)

We thank the reviewers for providing all the valuable and helpful comments on our manuscript. We have made substantial efforts to revise our paper based on these comments. The associated changes are highlighted in red color in the revised manuscript. The following are responses to the comments in order of occurrence.

> **1. The phrase "double-arm" is not commonly used and is very confusing. What is a double-arm study referring to? Both randomized trials and non-randomized trials can have double arms. Besides, randomized trials might have more than two arms. Clarifications are needed regarding whether it pertains to the randomized control trial that has two arms, but excludes trials that involve three or more comparators. To highlight the evidence strength of the pivotal trials, it would be better for the authors to use the term "randomized control trials" instead in this manuscript.**

We sincerely thank the referee for the valuable suggestion on the terminology used in the manuscript. We have replaced 'double-arm clinical trials' with 'randomized controlled trials.' Based on our understanding, 'double-arm clinical trials' typically refer to trials that have both an experimental group and a control group. However, the term 'double-arm' implies that these trials involve only two arms, which excludes trials with more than two arms (multi-arm trials), an oversight on our part. In our study, we included pivotal clinical trials that had an experimental group using the drug in our sample and a control group using its comparator, regardless of the number of arms. In cases where multi-arm trials had more than one experimental arm, we selected the arm that best aligned with the approved indication in the drug label. We thank the referee once again for pointing out that the term we initially used was not commonly accepted and accurate (*lines 126-130, page 5 in revised-track version*).

> **2. As mentioned in Table 2, the authors excluded Rituximab for the treatment of diffuse large-B-cell lymphoma in this analysis because the association was strongly influenced by this single outlier. Please provide some insights into the potential causes of such cases.**

We thank the referee for the insightful comment. We would like to discuss the potential causes of such cases from both price and clinical value perspectives.

Price. Rituximab was approved in China in 2000 but was only negotiated and included in the national reimbursement list in 2017, indicating a significant time gap between approval and listing. This

prolonged period can lead to changes in market conditions, with drug prices typically experiencing a downward trend over time post-launch. Unfortunately, we cannot provide the launch price or price changes of Rituximab in earlier years, as our data only covers recent years. To mitigate potential bias arising from this time gap, we conducted a sensitivity analysis in our original manuscript, excluding therapeutic indications that were launched before the implementation of price negotiation (2017). The sensitivity analysis confirmed the robustness of our findings.

Clinical value. In the pivotal clinical trial of Rituximab, the overall survival in the Rituximab group was 8.4 years, notably higher than the baseline survival of 3.5 years (which represents the survival in the control arm). This elevated baseline survival was in contrast to other therapeutic indications in our sample. Drugs for the treatment of cancers with longer baseline survival may exhibit greater added survival benefits. In our original manuscript, we examined the potential impact of cancer site on our results. In the revised manuscript, we have expanded our analysis to consider the potential influence of baseline survival on our findings, which confirmed the robustness of our findings. This additional analysis is based on the work conducted by Lauenroth et al. (2020) (*line 174, page 6 in revised-track version, appendix Table S4-6).*

In summary, factors related to the price and clinical value are potential causes of the outliers.

Reference

Lauenroth VD, Kesselheim AS, Sarpatwari A, Stern AD. Lessons from the impact of price regulation on the pricing of anticancer drugs in Germany. Health Aff (Millwood) 2020; 39(7): 1185-93

3. As most indications supported by single-arm trials were approved through conditional approval, the analysis of their clinical benefits was limited. The authors may consider including the results of confirmatory trials that the CDE requires for conditional approval indications, which might be available at the time of price negotiation.

We sincerely thank the referee for the suggestion. Out of the 27 therapeutic indications supported by single-arm clinical trials, 19 received conditional approval. To identify their confirmatory trials, we searched the CDE for review reports and drug labels. We found that only one indication (i.e., fluzoparib for the treatment of ovarian cancer) had available clinical outcomes from the confirmatory trial (FZPL-III-301-OC) at the time of negotiation (Table S1). However, it is important to note that this confirmatory trial supported the approval of a different indication for fluzoparib, not the indication approved based on the single-arm clinical trial. As a result, we excluded the confirmatory trial from the analysis of the indication supported by the single-arm clinical trial.

We fully agree with the referee that the evidence from single-arm clinical trials was limited. Nevertheless,

to facilitate the access of new drugs, regulators and payers are increasingly making approval and pricing & reimbursement decisions based on limited and uncertain clinical evidence, respectively. Our study's process of searching for clinical evidence underscores this prevailing trend in China. As shown in Table S1, a significant number of therapeutic indications supported by single-arm clinical trials were negotiated shortly after their launch. This means that the time period between indication approval and price negotiation was insufficient for generating confirmatory trial evidence.

Table S1 Availability of confirmatory trials for indications approved through conditional approval

No.	Generic name	Indication	Study ID	Availability	Year of negotiation	Indication approval year
1	Chidamide	peripheral T-cell lymphoma	NA	NA	2017	2014
2	Sintilimab	relapsed or refractory classical Hodgkin's lymphoma	NCT04044222	NA	2019	2018
3	almonertinib	EGFR T790M advanced or metastatic NSCLC	NCT03849768	NA	2020	2020
4	Denosumab	giant cell tumor of bone	NA	NA	2020	2019
5	Camrelizumab	relapsed or refractory classical Hodgkin's lymphoma	SHR-1210-III-CHL	NA	2020	2019
6	Camrelizumab	advanced hepatocellular carcinoma	SHR 1210-III-313	NA	2020	2020
7	Toripalimab	advanced or metastatic Melanoma	HMO-JS001-III-MM-01	NA	2020	2018
8	Tislelizumab	relapsed or refractory classical Hodgkin's lymphoma	BGB-A317-314	NA	2020	2019
9	Tislelizumab	advanced or metastatic urothelial bladder cancer	BGB-A317-310	NA	2020	2020
10	Zanubrutinib	mantle cell lymphoma	NA	NA	2020	2020
11	Zanubrutinib	chronic lymphocytic leukemia/small lymphocytic lymphoma	BGB-3111-304	NA	2020	2020
12	Orelabrutinib	mantle cell lymphoma	ICP-CL-00113	NA	2021	2020
13	Orelabrutinib	chronic lymphocytic leukemia/ small lymphocytic lymphoma	ICP-CL-00111	NA	2021	2020
14	Furmonertinib	EGFR T790M advanced or metastatic NSCLC	NA	NA	2021	2021
15	Pamiparib	advanced high-grade ovarian cancer	BGB-290-302	NA	2021	2021
16	Disitamab vedotin	advanced or metastatic gastric cancer	NA	NA	2021	2021
17	Fluzoparib	ovarian cancer	FZPL-III-301-OC	Available	2021	2020
18	Savolitinib	NSCLC	NCT04923945	NA	2022	2021
19	Carfilzomib	multiple myeloma	20170204	NA	2022	2021

Notes:NA, not available.

4. Some figures are not clearly presented. For example, the R^2 was not reported in Figures 3 and 4 nor in the legends; the y-axis of Figure 2 is confusing and needs an explanation in the legend.

We thank the referee for pointing out the concerns. In Figures 3 and 4, we intended to present the raw data to show the unadjusted relationship between treatment costs and clinical value. We did not report the R^2 for these figures as the data was presented without processing. To clarify our intention, we have emphasized in the revised manuscript that Figure 3 and Figure 4 presents the unadjusted association.

Instead, we reported the R^2 values in the regression analyses, which involved log-transforming treatment costs and excluding the outliers when necessary. In addition, we have revised Figure 2 as suggested and have checked all the figures and made necessary modifications (Figure 2 and Figure 3, *pages 18-19 in revised-track version*).

5. The authors mentioned that existing studies on the association between the prices and value of anticancer drugs, predominantly in the US and Europe with a few studies in Japan and China, demonstrated only weak or no association between prices and value, which are different from the results in this study. More comprehensive clarifications and interpretations for this are warranted. The statement "This implies that China may have achieved better performance than other countries in this regard" (on page 9, paragraph 2, lines 9-11), is not appropriate, even with the descriptions of limitations followed.

We would like to express our deepest appreciation to the referee for this invaluable comment. We have deleted the inappropriate statement and provided more clarifications and interpretations for the differences between China and the US and Europe (*lines 339-352, page 11 in revised-track version*):

Existing studies on the association between costs and clinical value of anticancer drugs have predominantly been conducted in the US and Europe, with a few studies in Japan and China, most of which demonstrated only weak or no association between prices and value (Del et al., 2017; Vokinger et al., 2021; Salas-Vega et al., 2020; Russo et al., 2021). However, sample and methodological differences across studies have hindered comparability (Russo et al., 2021). In particular, the primary differences related to the calculation of treatment costs may explain different conclusions observed among studies and countries (Russo et al., 2021). Additionally, the lack of international comparisons involving China using the same methodology and the same sample makes it difficult to assess the strength of the association estimates found in our findings sufficiently, future research is warranted to address this gap. Moreover, the impact of pricing-related policy on the association of costs and clinical value was under-researched. One study within the context of Germany revealed that price regulation in Germany had better aligned prices with clinical benefit, which was in line with our findings (Lauenroth et al., 2020).

Further research for other countries in this regard is encouraged.

References

Del Paggio JC, Sullivan R, Schrag D, et al. Delivery of meaningful cancer care: a retrospective cohort study assessing cost and benefit with the ASCO and ESMO frameworks. The Lancet Oncology 2017; 18(7): 887-94.

Vokinger KN, Hwang TJ, Daniore P, et al. Analysis of Launch and Postapproval Cancer Drug Pricing, Clinical Benefit, and Policy Implications in the US and Europe. JAMA Oncol 2021; 7(9): e212026.

Salas-Vega S, Shearer E, Mossialos E. Relationship between costs and clinical benefits of new cancer medicines in Australia, France, the UK, and the US. Soc Sci Med 2020; 258: 113042.

Russo P, Marcellusi A, Zanuzzi M, et al. Drug prices and value of oncology drugs in Italy. Value Health 2021; 24(9): 1273-8.

Zhang Y, Wei Y, Li H, et al. Prices and clinical benefit of national price-negotiated anticancer medicines in China. PharmacoEconomics 2022; 40(7): 715-24.

Lauenroth VD, Kesselheim AS, Sarpatwari A, Stern AD. Lessons from the impact of price regulation on the pricing of anticancer drugs in Germany. Health Aff (Millwood) 2020; 39(7): 1185-93.

6. The authors excluded indications supported by phase I clinical trials. Please provide the rationale. Phase I trials leading to drug approval also included efficacy endpoints like ORR, DOR. The evidence strength of pivotal phase I and phase II trials are similar.

We sincerely thank the referee for this insightful comment. We have revised the inclusion and exclusion criteria to include phase I clinical trials (*Figure 1, page 17 in revised-track version*). We initially excluded phase I clinical trials concerning that they primarily focus on safety and tolerability and involve fewer patients, whereas phase II clinical trials shift their focus to therapeutic efficacy and involve more patients. However, in this part, we overlooked the fact that decision-makers often base pricing and reimbursement decisions on limited data, including phase I clinical trials. We appreciate the referee for giving us the opportunity to refine our study design.

7. The manuscript contains multiple grammar errors. For example, in Figure 1, "1 indications" should be "1 indication"; on page 4, the last paragraph, line 4, "indications without …" should be "Indications without …". It is recommended to perform a comprehensive check and revision.

We thank the referee for careful reading. We have carefully and thoroughly checked and revised grammar errors in our manuscript.

8. According to the official website (https://www.cde.org.cn/)"CDE" is short for "Center for Drug Evaluation" instead of "Center of Drug Evaluation" shown in the manuscript.

We thank the referee for pointing it out. We have corrected it (*line 121, page 4, and appendix Table S1 in revised-track version*).

We wish the referee would be satisfied with our revision.

Reply to Reviewer 3's Comments for "Impact of price negotiation on the pricing of anticancer drugs in China" (PMEDICINE-D-23-01449R1)

We thank the reviewers for providing all the valuable and helpful comments on our manuscript. We have made substantial efforts to revise our paper based on these comments. The associated changes are highlighted in red color in the revised manuscript. The following are responses to the comments in order of occurrence.

> **1. Why is the association between prices of anticancer drugs and clinical value is important in the policy? The authors do not clearly explain this in the introduction part. The reduced association between prices of anticancer drugs and clinical value after price negotiation is expected because the NHSA needs to reduce it under the threshold.**

We thank the referee for the question. We have improved our expression in the introduction part to enhance the clarity and comprehensibility of our manuscript (*lines 88-93, page 3 and 4 in revised-track version*). In the context of limited resources, rationalizing the relationship between resource inputs and outcomes can enhance the efficiency of countries' healthcare systems. Therefore, optimizing the alignment of prices and clinical value has great potential to maximize health outcomes under resource constraints from a societal perspective, and incentivize the development of clinically meaningful drugs (Salas-Vega et al., 2016).

We also extended the explanation in the discussion part in the manuscript. The alignment of drug prices and value has great potential to benefit patients and health systems from two interrelated dimensions: accounting for the value of drugs and optimizing medical resource allocations (Fojo et al., 2016). The economic value of anticancer drugs should reflect the magnitude of health gain to justify price increases, and funds allocated to lower-value anticancer drugs should be redirected toward more valuable treatments to maximize health outcomes. For instance, drugs whose prices do not match their value are supposed to be subjected to lower prices during the negotiation process, which enables finite resources to be allocated towards treatments that offer patients greater clinical benefits.

We have carefully considered the the comment "the reduced association between prices of anticancer drugs and clinical value after price negotiation is expected because the NHSA needs to reduce it under the threshold". From our understanding, the association of prices and clinical value may not necessarily be linked to the NHSA's application of the ICER threshold. ICERs are employed to compare two treatments, and their applicability becomes challenging in assessments involving three or more treatments. ICER thresholds also raise concerns about health care rationing (Schnipper et al., 2015), and questions have been raised about whether drug treatment costs are related to their clinical value for

patients at a societal-level (Salas-Vega et al., 2016).

Our study compared different anticancer drugs' prices and clinical value at a societal-level. Three scenarios of the association between prices and clinical value may occur with the implementation of price negotiation: 1) unchanged; 2) reduced; and 3) enhanced. For example, in the third scenario, lower-value anticancer drugs experiencing larger price reductions (A to A') than higher-value drugs (B to B') would result in an enhanced association (Figure S1). In our study, we found that the implementation of price negotiation may not have significantly changed the association between prices and clinical value. One study on the impact of price regulation on the pricing of anticancer drugs in Germany showed that the implementation of price negotiation in Germany had enhanced the association between drug prices and clinical value (Lauenroth et al., 2020).

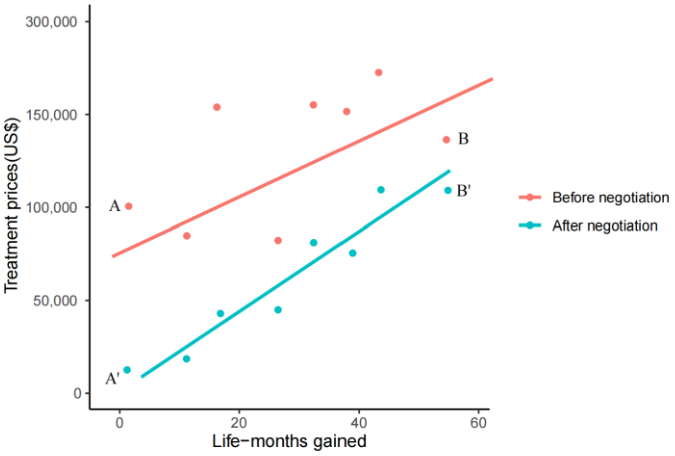

Figure S1. The third scenario of the association of prices and clinical value.

We thank the referee for sharing the perspective, and we hope we understood it correctly and explained our perspective clearly.

References

Schnipper LE, Davidson NE, Wollins DS, et al. American Society of Clinical Oncology Statement: A conceptual framework to assess the value of cancer treatment options. 2015; 33(23): 2563-77.

Salas-Vega S, Shearer E, Mossialos E. Relationship between costs and clinical benefits of new cancer medicines in Australia, France, the UK, and the US. Soc Sci Med 2020; 258: 113042.

Fojo T, Lo AW. Price, value, and the cost of cancer drugs. The Lancet Oncology 2016; 17(1): 3-5.

Lauenroth VD, Kesselheim AS, Sarpatwari A, Stern AD. Lessons from the impact of price regulation on the pricing of anticancer drugs in Germany. Health Aff (Millwood) 2020; 39(7): 1185-93

2. The interpretations in Figure 2 and figure 3 are not clear. What do these two figures imply?

We thank the referee for the constructive comment. We have modified the interpretations of Figure 2 and Figure 3 and more details were provided in Figure legends (*Figure 2 and Figure 3, pages 18-19 in*

revised-track version).

In Figure 2, we primarily compared the treatment costs between two groups: those before negotiation and those after negotiation. Additionally, we compared treatment costs between indications supported by randomized controlled trials and those supported by single-arm clinical trials, both before and after price negotiation. We attached asymptotic p values in Figure 2 to show group differences, making these comparisons more straightforward. In Figure 3, we showed the unadjusted association between costs and added life-months gained of indications supported by randomized controlled trials.

3. In figure 3, there is clearly reduced association between prices of anticancer drugs and clinical value after price negotiation. But in Table 2, the coefficients remain largely unchanged. Any reasons?

We thank the referee for the insightful comment. In Figure 3, we intended to present the raw data of all included indications supported by randomized controlled trials to illustrate the unadjusted relationship between treatment costs and clinical value (life-months gained), so we did not account for data distribution and did not exclude the extreme outliers. This was in reference to the work by Lauenroth et al. (2020). To clarify our intention, we have emphasized in the revised manuscript that Figure 3 presents the unadjusted association. However, we excluded the outliers and log-transformed the treatment costs based on the data distribution when we conducting regression analyses (Table 2 in the original and revised manuscript). This explains the observed difference between Figure 3 and Table 2.

Following the comment, we additionally conducted a reanalysis of the data displays in Figure 3. First, due to the skewed distribution of treatment costs, we log-transformed the data to approximately conform to normality. Second, we excluded the outliers. The data processing procedure used here is aligned with the regression analyses shown in Table 2 in the manuscript. The following plot (Figure S2) displays the results of the reanalysis. The regression lines in Figure S2 shows the adjusted relationship between log-transformed treatment costs and clinical value (life-months gained), which is in consistent with the relationship shown in Table 2 in the manuscript.

Reference

Lauenroth VD, Kesselheim AS, Sarpatwari A, Stern AD. Lessons from the impact of price regulation on the pricing of anticancer drugs in Germany. Health Aff (Millwood) 2020; 39(7): 1185-93

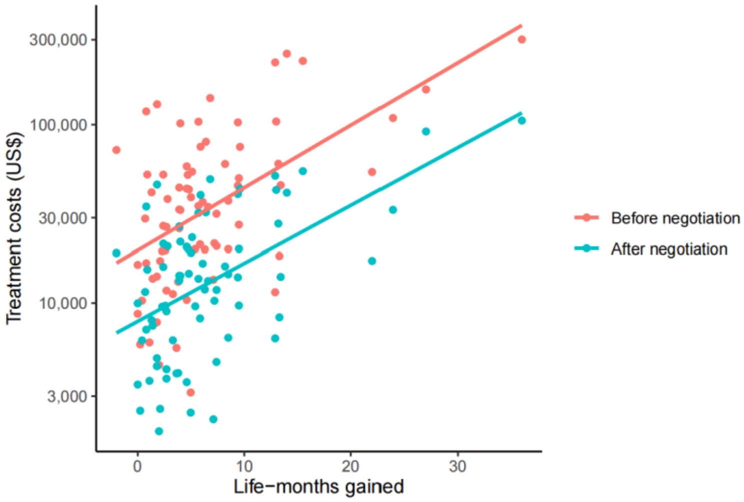

Figure S2. The adjusted association between treatment costs and added life-months gained of indications supported by randomized controlled trials in China.

Notes: The lines in the plot represents the relationship between log (treatment costs) and life-months gained after excluding the outliers. Of note, the scale for treatment costs (Y-axis) has been log-transformed, but the axis labels display the original values for a clearer visual representation.

4. The implications of findings for other countries are a bit weak. The authors need to strengthen this part.

Many thanks for this valuable comment. We have strengthened the implications of our findings for other countries (*lines 329-337, page 10 in revised-track version*). The accomplishments in China in the regard hold substantial potential for offering valuable insights into drug price regulation, not only for other low- and middle-income countries grappling with resource constraints and escalating drug expenditures but also for high-income nations. The fact that high-expenditure drugs will be subjected to price negotiation in the US under the Inflation Reduction Act of 2022 highlights the increasing recognition of the significant influence that price negotiation can exert. In this context, China serves as an example of how price negotiation can be designed to better align prices with clinical value in addition to reducing drug prices.

5. The writing of this article should be strengthened.

We thank the referee for careful reading. We have carefully and thoroughly polished the English language in our manuscript.

We wish the referee would be satisfied with our revision.

Reply to Reviewer 4's Comments for "Impact of price negotiation on the pricing of anticancer drugs in China" (PMEDICINE-D-23-01449R1)

We thank the reviewers for providing all the valuable and helpful comments on our manuscript. We have made substantial efforts to revise our paper based on these comments. The associated changes are highlighted in red color in the revised manuscript. The following are responses to the comments in order of occurrence.

1. Please provide line number for easier reference.

We thank the referee for the suggestion. We have added line number in the revised manuscript.

2. Introduction: The introduction mentions various policy instruments in pricing and reimbursement, such as external price referencing (EPR), managed entry agreements (MEA), and health technology assessment (HTA), but does not delve into how these differ from or complement the reimbursement-linked price negotiation strategy in China. A brief comparative insight into these methods could help set the stage for why the Chinese approach is unique or noteworthy.

We thank the referee for the insightful comment. We have described how China embedded these policy instruments in the price negotiation in the revised manuscript *(lines 68-72, page 3 in the revised-track version)*, and details can also be found in the paragraph below.

To curtail drug prices, ensure affordable patient access, and safeguard the sustainability of health care systems, health authorities use a mix of policy instruments in pricing and reimbursement. In addition to external price referencing (EPR), managed entry agreements (MEA), and health technology assessment (HTA), value-based pricing and strategic procurement are being explored internationally including in China (Vogler S et al., 2018; Kaltenboeck et al., 2018; Ferrario et al., 2017; Nguyen et al., 2015). In China, such reforms were formally introduced in the form of reimbursement-linked drug price negotiation in 2017. Since then, health authorities have been negotiating prices for innovative drugs directly with pharmaceutical companies annually, informed by HTA and accompanied by the EPR and MEA, trying to realize value-based strategic purchase of medical insurance (Tang et al., 2020; Liu et al., 2022).

References

Vogler S, Paris V, Panteli D. Ensuring access to medicines: How to redesign pricing, reimbursement and procurement?: World Health Organization, Regional Office for Europe; 2018.

Kaltenboeck A, Bach PB. Value-Based Pricing for Drugs: Theme and Variations. JAMA 2018; 319(21):

2165-6.
Ferrario A, Humbert T, Kanavos P, Pedersen HB. Strategic procurement and international collaboration to improve access to medicines. Bulletin of the World Health Organization 2017; 95(10): 720-2.
Nguyen TA, Knight R, Roughead EE, Brooks G, Mant A. Policy options for pharmaceutical pricing and purchasing: issues for low- and middle-income countries. Health Policy Plan 2015; 30(2): 267-80.
Tang M, Song P, He J. Progress on drug pricing negotiations in China. Biosci Trends 2020; 13(6): 464-8.
Liu GG, Wu J, He X, Jiang Y. Policy Updates on Access to and Affordability of Innovative Medicines in China. Value Health Reg Issues 2022; 30: 59-66.

3. Introduction: While the introduction hypothesizes that price negotiation has led to drug pricing being more aligned with clinical value in China, it doesn't specify what measures or metrics will be used to assess 'clinical value.' Providing a preview of these metrics in the introduction could help readers better understand the scope and methodology of the research, thereby setting clearer expectations for what will be covered in the subsequent sections.

We sincerely thank the referee for this valuable comment. We have added the metrics in the introduction to specify that the clinical value including patients' survival, quality of life, and safety (*lines 101-103, page 4 in revised-track version*).

4. Method, Sample selection: Why did the authors exclude indications specifically for pediatric use? Providing an explanation for this decision could be helpful. Additionally, the inclusion of indications that were withdrawn from the NRDL raises questions. Logically, if an indication or drug is removed from the NRDL, it suggests that the price negotiation was unsuccessful, and therefore, it should not impact the drug's price. Could the authors clarify the rationale behind including these withdrawn indications in the study?

We thank the referee for the questions. Regarding the first question, we excluded the indication for pediatric use in our study for two reasons: 1) we only identified one indication for pediatric use in our study, the exclusion of this indication could mitigate its difference compared to other indications for adult use, ensuring the consistency of our sample, and 2) pediatric dosages often differ from adult dosages, possibly biasing the cost analysis and the relationship between the costs and clinical value. The indication excluded was Pegaspargase for the treatment of acute lymphocytic leukemia in children, with a recommended dose of 2500 IU/m2 once every 14 days. In our study, treatment costs over expected treatment durations were calculated based on drug prices and dosing information. Since the body surface area of children is smaller than that of adults, the treatment costs for children are likely to be lower than that for adults. Therefore, including indications for pediatric use may introduce bias into the costs

analysis and the relationship between treatment costs and clinical value. We did not provide a detailed explanation in the manuscript because our methodology for calculating treatment costs was presented below the 'sample selection' section. To avoid confusion, we revised our expression as "we also excluded one indication for pediatric use to mitigate the difference among indications and ensure the consistency of our sample" (*lines 111-113, page 4 in revised-track version*).

To enhance clarity regarding the issue raised in the second question, we have revised our expression as "indications that were withdrawn from the NRDL but had previously been listed through price negotiation were included, since the negotiated prices were reached at the time of price negotiation and were publicly available" (*lines 113-116, page 4 in revised-track version*). Our targeted sample consisted of anticancer drugs along with their indications that underwent price negotiation successfully between 2017 and 2022. Despite the possibility of these indications being withdrawn from the NRDL after their inclusion through price negotiation, they did undergo price negotiation successfully, resulting in agreed-upon prices between pharmaceutical companies and the National Healthcare Security Administration (NHSA). Furthermore, indications being withdrawn from the NRDL does not necessarily mean the price negotiation at the time was unsuccessful. The withdrawal decision was made based on the specific situation at the time of withdrawal, possibly because better alternatives had emerged after the price negotiation or other reasons. For instance, Lapatinib, for the treatment of HER-2-positive advanced breast cancer, was negotiated in 2017 and listed for reimbursement in the NRDL from 2017 to 2021. In 2021, Lapatinib was withdrawn from the NRDL because of the failed re-negotiation. In such a case, the failed re-negotiation in 2021 could not suggest that the price negotiation conducted in 2017 was unsuccessful.

We wish we understood the concerns raised by the referee correctly and have provided our rationale for including the withdrawn indication clearly.

5. Method, Data sources and extraction: Instead of relying on single clinical trials for each drug and indication, it may be more robust to utilize results from systematic reviews or to conduct a meta-analysis that synthesizes all available evidence. This approach could provide a more comprehensive and reliable understanding of a drug's impact on a specific indication.

6. Method, data sources: "The extracted data were limited to those available at the time of price negotiation." I can understand why the authors did that, but I still think it would be better to use all evidence available (even after the negotiation) to evaluate the effectiveness/efficacy of the drug.

We thank the referee for the two comments. Since the two comments were highly related, we would like to merge them together to address the concerns raised by the referee. We fully agree with the referee that

systematic reviews or meta-analyses could provide more robust evidence of the effectiveness/efficacy of the drug or the indication and we appreciate the referee for understanding why we limited the evidence at the time of negotiation. Following the comments, we have made efforts to find the evidence from systematic reviews or meta-analyses at the time of negotiation. At first, we would like to share our perspective regarding why we chose the evidence available at the time of negotiation and did not consider the evidence generated after negotiation.

We chose the evidence available at the time of negotiation because we believe that the method and data type chosen in studies should primarily serve the research purpose. For this study, our objective was to investigate the impact of price negotiation on the pricing of anticancer drugs, providing policy implications for China. Regulators and payers are increasingly making approval and pricing & reimbursement decisions based on limited and uncertain clinical evidence, respectively. Choosing the clinical evidence at the time of negotiation was basically a reflection of the real-world situation, which can help explain how the decisions were made and help researchers and policymakers explore whether these decisions made by the payers were rational, ultimately offering valuable insights from the experience of price negotiation implementation. At the moment of decision-making, payers are not able to access future evidence, and decisions have to be made based on existing evidence. Under such a context, the evidence available at the time of negotiation was chosen to align with the study's purpose and reflect real-world decision-making, which is consistent with other related studies (Vivot et al., 2017; Russo et al., 2021).

The incorporation of high-level evidence after negotiation could enhance the strength of the evidence. However, this approach would diminish the policy implications of this study. Using evidence after price negotiation to evaluate the impact of price negotiation on the relationship between prices and the clinical value of anticancer drugs in China could introduce bias. This is because the impact would result from both the price negotiation itself and changes in clinical value after negotiation. The changes in clinical value after negotiation may affect the relationship between prices and clinical value at the time of negotiation, making post-negotiation evidence less suitable and reliable for assessing the policy effects of price negotiation. We hope the referee would understand our perspective.

Second, we would like to provide the methods and results of our attempt to find evidence from systematic reviews or meta-analyses at the time of negotiation.

Methods

We searched PubMed for publications prior to price negotiation using the following search terms: (drug name [Title/Abstract]) AND (systematic review [Title/Abstract] OR meta-analysis [Title/Abstract]). We included systematic reviews or meta-analyses that met the following criteria:

(1) The targeted population should align with the approved indication, including histology, tumor type, prior interventions, prespecified risk factors/biomarkers, tumor stage, treatment line, required use in combination, and the type of combination (Trotta et al., 2011). This criterion was established taking into account that limited and uncertain clinical evidence often leads regulatory agencies to make

approval decisions more conservatively. In many cases, the histology, type of tumor, prior interventions, prespecified risk factors/biomarkers, tumor stage, treatment line, required use in combination, and type of combination are specifically defined for a particular indication (Trotta et al., 2011; Mintzes et al., 2019). Furthermore, to ensure the sustainability of medical funds, the National Healthcare Security Administration (NHSA) in China has implemented restrictions on reimbursable indications. This means that only the specific patient populations defined in the NHSA-approved indications are eligible for reimbursement, and not every indication for a given drug will be covered.

(2) The overall survival or progression-free survival of the drug in relative to its control group(s) was presented in months. This criterion was chosen because, in our study, the measurement of survival as one of the clinical value indicators was expressed in months, consistent with other studies (Salas-Vega et al., 2020; Lauenroth et al., 2020).

(3) The systematic review or meta-analysis for the specific indication must include its pivotal clinical trial.

Results

Out of the 76 indications supported by randomized controlled clinical trials, we only identified 25 indications that had possible related published systematic reviews or meta-analyses at the time of negotiation. After reading the full text of these publications, we found that the publications for the 24 indications were not compatible with our study design in terms of fulfilling the three criteria. That is to say, only one indication (i.e., axitinib for the treatment of metastatic renal cell carcinoma) had available systematic review and meta-analysis at the time of negotiation that aligned with our study design. However, this systematic review and meta-analysis derived its overall survival data from one single clinical trial, which coincided with the pivotal clinical trial we included for our analysis.

Table S1 provides the details of the searching results. As shown in Table 1, the main reasons for exclusion include: 1) the target population was not aligned with the indication in terms of the prior interventions used, the use in combination required, and the type of combination; 2) overall survival or progression-free survival was not presented in months; and 3) the systematic review or meta-analysis of the indication did not include the corresponding pivotal clinical trial.

The following reasons could explain the results of this attempt. First, the heterogeneity embedded in the study populations of systematic reviews or meta-analyses contradicts the intention of the regulatory agency and the NHSA to specify and restrict the targeted population, including the aforementioned histology, prior interventions, prespecified risk factors/biomarkers, tumor stage, use in combination required, and type of combination. Second, systematic reviews or meta-analyses often report hazard ratios for overall survival and progression-free survival, while data on overall survival and progression-free survival in months are not commonly available. Third, recent regulatory reforms in China have led

to a significant increase in the approval of anticancer drugs (Liu et al., 2022). Decision-makers are increasingly making approval decisions based on limited and uncertain clinical evidence. The NHSA is also placing greater emphasis on strengthening the connection between price negotiation and the review and approval process. Consequently, a considerable proportion of anticancer drugs did not have supporting systematic reviews or meta-analyses available at the time of price negotiation, because there was not enough time to generate high-level evidence.

It should be noted that we did not conduct systematic reviews or meta-analyses since the systematic reviews or meta-analyses conducted by the authors would not represent the evidence reviewed by the NHSA. Furthermore, we consulted a senior expert deeply involved in the price negotiation process, who confirmed that the experts from the NHSA usually would not conduct systematic reviews or meta-analyses for candidate drugs to be negotiated and the available evidence of the systematic reviews or meta-analyses at the time of negotiation were published materials. This again illustrates the rationale for searching for evidence from published systematic reviews and meta-analyses at the time of negotiation.

All in all, we appreciate the reviewer's input to make this study more scientifically rigorous, though systematic reviews or meta-analyses were not very compatible with our study design. We think this is probably why existing related impactful studies had to rely on single clinical trials for each drug and indication (Vokinger et al., 2020; Vokinger et al., 2021; Vivot et al., 2017; Del et al., 2017).

References

Vivot A, Jacot J, Zeitoun JD, Ravaud P, Crequit P, Porcher R. Clinical benefit, price and approval characteristics of FDA-approved new drugs for treating advanced solid cancer, 2000-2015. Ann Oncol 2017; 28(5): 1111-6.

Russo P, Marcellusi A, Zanuzzi M, et al. Drug Prices and Value of Oncology Drugs in Italy. Value Health 2021; 24(9): 1273-8.

Lauenroth VD, Kesselheim AS, Sarpatwari A, Stern AD. Lessons from the impact of price regulation on the pricing of anticancer drugs in Germany. Health Aff (Millwood) 2020; 39(7): 1185-93.

Salas-Vega S, Shearer E, Mossialos E. Relationship between costs and clinical benefits of new cancer medicines in Australia, France, the UK, and the US. Soc Sci Med 2020; 258: 113042.

Trotta F, Leufkens HG, Schellens JH, Laing R, Tafuri G. Evaluation of oncology drugs at the European Medicines Agency and US Food and Drug Administration: when differences have an impact on clinical practice. J Clin Oncol 2011; 29(16): 2266-72.

Mintzes B, Vitry A. Flawed evidence underpins approval of new cancer drugs. 2019; 366: l5399.

Liu Y, Zhang N, Xie C, et al. Evolution of drug regulations and regulatory innovation for anticancer drugs in China. Acta pharmaceutica Sinica B 2022; 12(12): 4365-77.

Vokinger KN, Hwang TJ, Grischott T, et al. Prices and clinical benefit of cancer drugs in the USA and Europe: a cost–benefit analysis. The Lancet Oncology 2020; 21(5): 664-70.

Vokinger KN, Hwang TJ, Daniore P, et al. Analysis of Launch and Postapproval Cancer Drug Pricing,

Clinical Benefit, and Policy Implications in the US and Europe. JAMA Oncol 2021; 7(9): e212026.
Del Paggio JC, Sullivan R, Schrag D, et al. Delivery of meaningful cancer care: a retrospective cohort study assessing cost and benefit with the ASCO and ESMO frameworks. The Lancet Oncology 2017; 18(7): 887-94.

Table S1. The results of identifying the systematic reviews or meta-analyses for indications supported by randomized control trials.

No.	Generic name	Indication	Negotiation year	Possible related publications	Main reason for exclusion
1	Abiraterone	metastatic castration-resistant prostate cancer	2017	(Zhi-Rui et al., 2014) Abiraterone for treatment of metastatic castration-resistant prostate cancer: a systematic review and meta-analysis	no data in months
2	Erlotinib	EGFR mutation-positive advanced NSCLC (maintenance treatment)	2017	(Jian Zhang et al., 2012) Maintenance erlotinib improves clinical outcomes of unresectable advanced non-small cell lung cancer: A meta-analysis of randomized controlled trials	no data in months
3	Erlotinib	EGFR mutation-positive advanced NSCLC (previously treated)	2017	（Hu Ma et al., 2016）The efficacy of erlotinib versus conventional chemotherapy for advanced nonsmall-cell lung cancer<br>（J. L. Xu et al., 2015）Chemotherapy plus erlotinib versus chemotherapy alone for treating advanced non-small cell lung cancer: a Meta-analysis	no data in months
4	Erlotinib	EGFR mutation-positive advanced NSCLC (first-line)	2017	（Hu Ma et al., 2016）The efficacy of erlotinib versus conventional chemotherapy for advanced nonsmall-cell lung Cancer<br>（J. L. Xu et al., 2015）Chemotherapy plus erlotinib versus chemotherapy alone for treating advanced non-small cell lung Cancer: a Meta-analysis	no data in months
5	Lapatinib	HER-2-positive advanced breast cancer	2017	(Tobias Engel et al., 2013) Lapatinib plus chemotherapy or endocrine therapy (CET) versus CET alone in the treatment of HER-2-overexpressing locally advanced or metastatic breast cancer: systematic review and meta-analysis	no data in months
6	Lenalidide	multiple myeloma	2017	(Shu-Kai et al., 2015) Efficacy and safety of lenalidomide in the treatment of multiple myeloma: a systematic review and meta-analysis of randomized controlled trials	no data in months

No.	Generic name	Indication	Negotiation year	Possible related publications	Main reason for exclusion
7	Rituximab	Diffuse Large-B-Cell Lymphoma (previously untreated)	2017	(Xuan Zhou et al., 2017) Rituximab maintenance therapy for patients with diffuse large B-cell lymphoma: A meta-analysis	prior interventions; no data in months
8	Bortezomib	multiple myeloma (first-line, in combination with melphalan and prednisone)	2017	(Kathleen et al., 2016) Bortezomib for the treatment of multiple myeloma	prior interventions; type of combination required; no data in months
9	Bortezomib	relapsed multiple myeloma (monotherapy)	2017	(Kathleen et al., 2016) Bortezomib for the treatment of multiple myeloma	prior interventions; use in combination required; no data in months
10	Trastuzumab	HER2 positive metastatic breast cancer (in combination with paclitaxel or docetaxel)	2017	(Zhi-Qiao et al., 2017) Efficacy and safety of lapatinib and trastuzumab for HER2-positive breast cancer: a systematic review and meta-analysis of randomised controlled trials<br>(Zhen-Li 2013) Efficacy and safety of Trastuzumab added to standard treatments for HER2-positive metastatic breast cancer patients	type of combination required; no data in months
11	Sorafenib	advanced hepatocellular carcinoma	2017	(Songlin et al., 2014) An Updated Meta-analysis of randomized controlled trials assessing the effect of sorafenib in advanced hepatocellular carcinoma	no data in months
12	Sorafenib	thyroid cancer	2017	(Ligy et al., 2014) Sorafenib in metastatic thyroid cancer: a systematic review	not including the pivotal trial
13	Afatinib	advanced or metastatic NSCLC with EGFR mutations (did not receive EGFR tyrosine kinase inhibitor (TKI) treatment)	2018	(Zhang Y et al., 2017) The efficacy and toxicity of afatinib in advanced EGFR-positive non-small-cell lung cancer patients after failure of first-generation tyrosine kinase inhibitors: a systematic review and meta-analysis.	prior interventions

No.	Generic name	Indication	Negotiation year	Possible related publications	Main reason for exclusion
14	Axitinib	advanced RCC	2018	(Hai Wang et al., 2016) Comparative efficacy and safety of axitinib versus sorafenib in metastatic renal cell carcinoma: a systematic review and meta-analysis	**could be included (only one study had OS data, which was the pivotal study)**
15	Azacitidine	myelodysplastic syndromes	2018	(Ronit Gurion et al., 2010)5-azacitidine prolongs overall survival in patients with myelodysplastic syndrome - a systematic review and meta-analysis	no data in months
16	Crizotinib	ALK-Positive Advanced NSCLC	2018	(Hao Hu et al., 2016) Is there a benefit of first- or second-line crizotinib in locally advanced or metastatic anaplastic lymphoma kinase-positive non-small cell lung cancer? a meta-analysis	not including the pivotal trial; no data in months
17	Pazopanib	advanced RCC (first-line and patients who have received cytokine therapy)	2018	(Victor C et al., 2016) Pazopanib as a second-line treatment for non-cytokine-treated metastatic renal cell carcinoma: a meta-analysis of the effect of treatment	treatment line; no data in months
18	Regorafenib	advanced gastrointestinal stromal tumours	2018	(Zhenan Zhang et al., 2017) Efficacy and safety of regorafenib for advanced gastrointestinal stromal tumor after failure with imatinib and sunitinib treatment	did not report the survival of regorafenib in relative to the control group
19	Sunitinib	metastatic RCC	2018	(B Gyawali et al., 2017) Adjuvant sunitinib for high-risk-resected renal cell carcinoma: a meta-analysis of ASSURE and S-TRAC trials<br>(Miriam et al., 2015) Sunitinib in metastatic renal cell carcinoma: a systematic review of UK real world data	not including pivotal clinical trial; tumor stage
20	Cetuximab	metastatic colorectal cancer (in combination with irinotecan)	2018	(L I Lin et al., 2016) Efficacy of cetuximab-based chemotherapy in metastatic colorectal cancer according to RAS and BRAF mutation subgroups: A meta-analysis	type of combination required; no data in months

No.	Generic name	Indication	Negotiation year	Possible related publications	Main reason for exclusion
21	Alectinib	advanced ALK-positive NSCLC	2019	(Junsheng et al., 2018) The efficacy and safety of alectinib in the treatment of ALK+ NSCLC: a systematic review and meta-analysis	not including pivotal clinical trial
22	Olaparib	platinum-sensitive, relapsed ovarian cancer or primary peritoneal cancer	2019	(Jiao Ma et al., 2019) Efficacy and safety of olaparib maintenance therapy in platinum-sensitive ovarian cancer patients with BRCA mutations: a meta-analysis on randomized controlled trials	no data in months
23	Eribulin	metastatic breast cancer	2021	(Chris Twelves et al., 2014) Efficacy of eribulin in women with metastatic breast cancer: a pooled analysis of two phase 3 studies	prior interventions
24	Daratumumab	multiple myeloma (in combination with lenalidomide and dexamethasone)	2021	(Yin Wang et al., 2021) Efficacy and safety of daratumumab in the treatment of multiple myeloma: a systematic review and meta-analysis	type of combination required
25	Daratumumab	multiple myeloma (in combination with bortezomib and dexamethasone)	2021	(Yin Wang et al., 2021) Efficacy and safety of daratumumab in the treatment of multiple myeloma: a systematic review and meta-analysis	type of combination required

\

7. Method, Statistical analysis: ". whether price negotiation has led to a reduction in the variation of drug prices for a specific value… here Y represents treatment costs and X represents clinical value.". So, did you examined price variation or treatment costs variation, I think they are different metrics.

We deeply thank the referee for pointing out the confusion we have caused. We examined the treatment costs variation and have revised the expression accordingly (*line 200, page 7 in revised-track version*).

8. Results, Figure 3: Upon examining Figure 3, I have concerns about data sparsity at the higher end of the life-months gained spectrum. In situations like this, a linear model could be unduly influenced by a few outlier data points on the right-hand side of the plot, potentially skewing the results.

We appreciate the referee's insightful comment. Indeed, the results of linear regressions could be influenced by outliers. In Figure 3, we intended to present the raw data of all included indications supported by randomized controlled trials to illustrate the unadjusted relationship between treatment costs and clinical value (life-months gained). Therefore, we did not account for data distribution and did not exclude the extreme outliers on the right side of the plot. This approach was in reference to the work by Lauenroth et al. (2020). In the revised manuscript, we have explicitly stated that Figure 3 shows the unadjusted association. Notably, in our regression analyses (both in the original and revised manuscript, as shown in Table 2), we excluded the outliers on the right-hand side of the plot and log-transformed the treatment costs based on the data distribution for statistical robustness.

To address the concerns raised by the referee, we conducted a reanalysis of the data presented in Figure 3. The results of this reanalysis are displayed in Figure S1 to Figure S4 below. In Figure S1, which shows the raw data of treatment costs and life-months gained without any data processing, we observed data sparsity and a few outliers at the higher end of the life-months gained spectrum.

To account for data sparsity and the skewed distribution of treatment costs, we log-transformed the treatment costs and then plotted the scatter of life-months gained against log-transformed treatment costs in Figure S2. Figure S2 demonstrates a much better data density compared to Figure S1.

Next, to address the potential influence of outliers on the results of linear regressions, we excluded the outliers on the right-hand side of the plot and analyzed the relationship between log-transformed treatment costs and life-months gained, as shown in Figure S3 and columns (1) and (2) of Table S1. It's worth noting that the data processing procedure used in Figure S3 is consistent with the regression analyses presented in Table 2 of the manuscript.

To examine whether the outliers significantly influenced the results of the regression analyses, we also

conducted regression analysis that included the outliers, as illustrated in Figure S4 and columns (3) and (4) of Table S1. The relationship between log-transformed treatment costs and life-months gained remains largely unchanged compared to regression analysis excluding the outliers. A comparison of the results from regression analyses, including and excluding the outliers, confirmed the robustness of our findings.

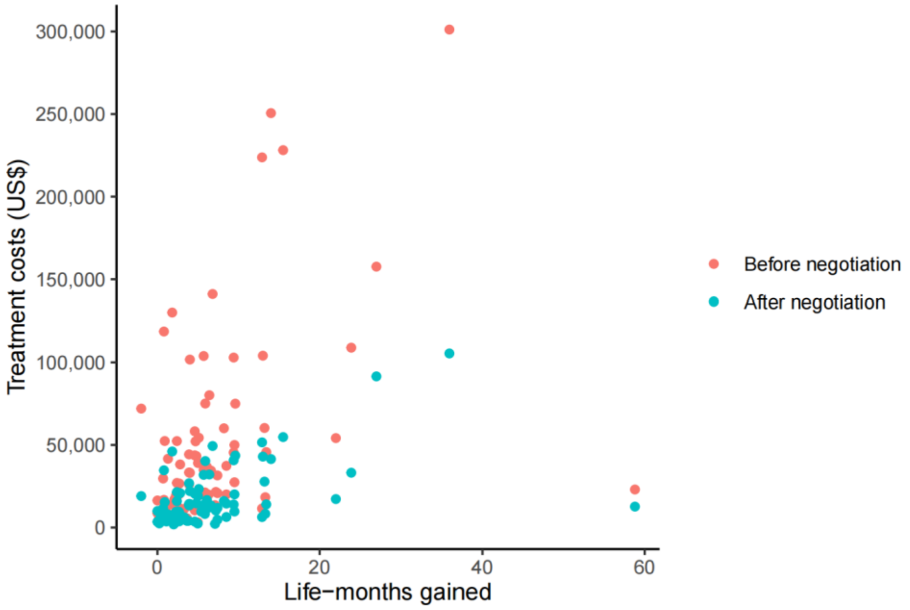

Figure S1. Treatment costs and added life-months gained of indications supported by randomized controlled trials in China

Note: Data included all indications supported by randomized controlled trials in the sample.

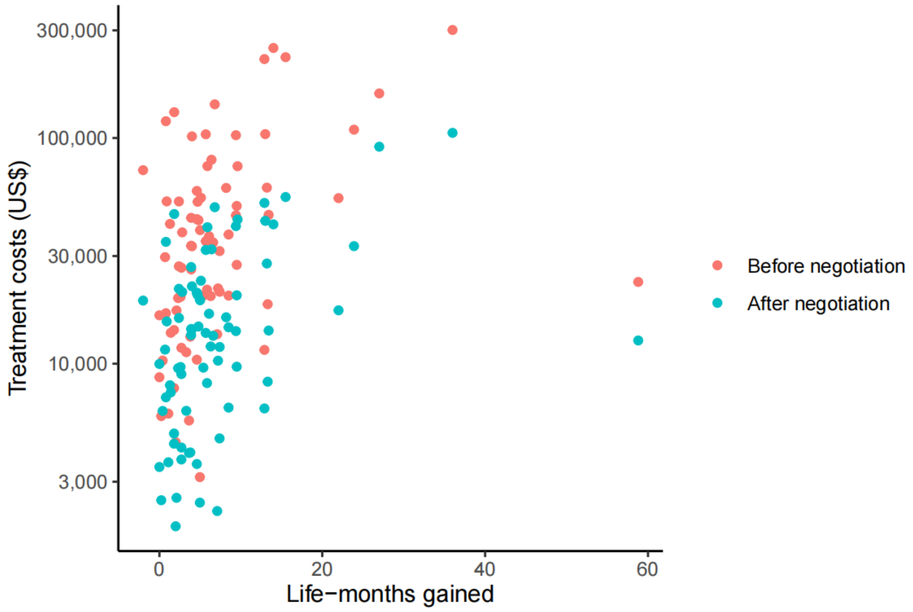

Figure S2. Treatment costs and added life-months gained of indications supported by randomized controlled trials in China.

Notes: Data includes all indications supported by randomized controlled trials in the sample. Of note, the scale for treatment costs (Y-axis) has been log-transformed, but the axis labels display the original values

for a clearer visual representation.

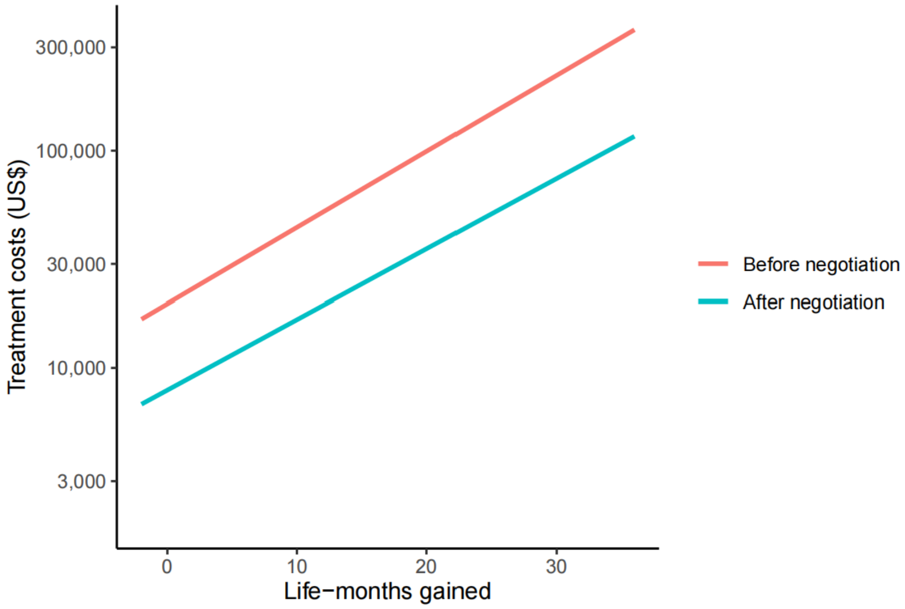

Figure S3. The relationship between treatment costs and added life-months gained of indications supported by randomized controlled trials in China.

Notes: **Data excluded the extreme outliers.** Of note, the scale for treatment costs (Y-axis) has been log-transformed, but the axis labels display the original values for a clearer visual representation.

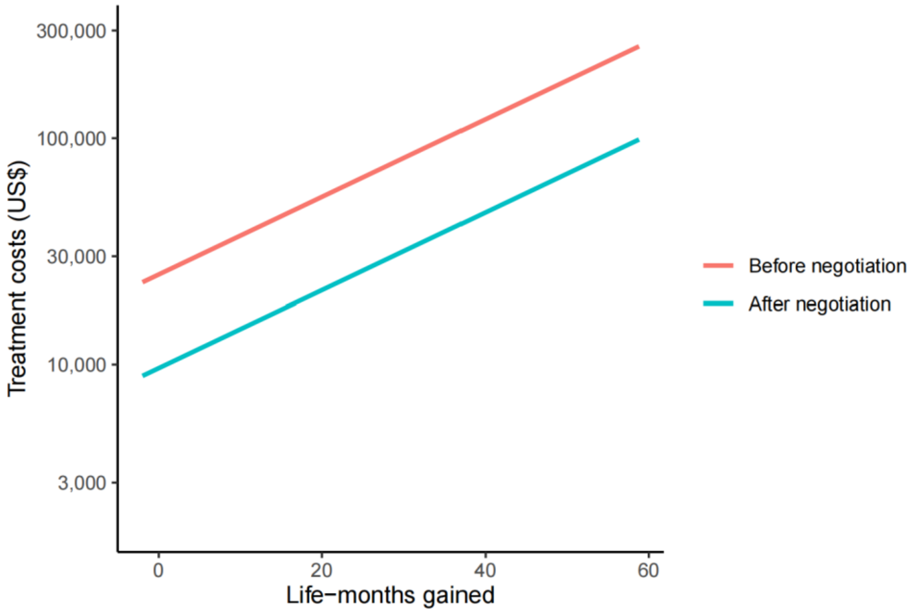

Figure S4. The relationship between treatment costs and added life-months gained of indications supported by randomized controlled trials in China.

Notes: **Data included the extreme outliers.** Of note, the scale for treatment costs (Y-axis) has been log-transformed, but the axis labels display the original values for a clearer visual representation.

Table S1. Regression analyses of log-transformed treatment costs and life-months gained

Variable	**Model with data excluding the outliers**		**Model with data including the outliers**	
	(1)	(2)	(3)	(4)
Dependent variables	Log (costs before negotiation)	Log (costs after negotiation)	Log (costs before and negotiation)	Log (costs after and negotiation)
Life-months gained	0.035***	0.032***	0.017**	0.017***
	(0.007)	(0.006)	(0.005)	(0.005)
Constant	4.295***	3.897***	4.397***	3.984***
	(0.060)	(0.056)	(0.060)	(0.055)
Observations	75	75	76	76
R^2	0.276	0.274	0.124	0.145
Adj R^2	0.266	0.264	0.113	0.133

Notes: Standard errors (SE) are provided in parentheses. ***: $p \leqslant 0.001$, **: $p \leqslant 0.01$, *: $p \leqslant 0.05$.

In summary, Figure 3 was designed to display the raw data and illustrate the unadjusted relationship between treatment costs and life-months gained. To address the data sparsity and outliers, we opted to exclude the outliers found on the right-hand side of the plot and log-transformed treatment costs when conducting the regression analyses (as detailed in Table 2 of both the original and revised manuscripts). Importantly, our results remained robust even when these outliers were included in the regression analyses.

Reference

Lauenroth VD, Kesselheim AS, Sarpatwari A, Stern AD. Lessons from the impact of price regulation on the pricing of anticancer drugs In Germany. Health Aff (Millwood) 2020; 39(7): 1185-93.

We wish the referee would be satisfied with our revision and explanation.
