# Supplementary material for: Price negotiation and pricing of anticancer drugs in China: An observational study
Source: PLoS Med. 2024 Jan 2;21(1):e1004332. doi: 10.1371/journal.pmed.1004332 (PMC10793910; doi:10.1371/journal.pmed.1004332)
Supplement: Attachment — Submitted filename: 231206-Response to comments.docx [file pmed.1004332.s015.docx]

Reply to Academic Editor’s Comments for "Impact of price negotiation on the pricing of anticancer drugs in China" (PMEDICINE-D-23-02974R1)

We appreciate the editor for the valuable and helpful comments on our manuscript. We have revised the manuscript accordingly, highlighting the changes in red in the revised manuscript. The following responses address the comments in order of occurrence.

**For me, Tables 2 and 3 were very hard to follow -- they seem like the kind of tables that belong in an economics journal, not PLOS.**

We appreciate the editor's valuable feedback. In response to the comment, we have revised Tables 2 and 3, along with relevant tables in the supporting information, to enhance clarity (*pages 24-26 in revised-track version*).

**Why do Figs 3 and 4 highlight the unadjusted association -- those Figures make it seem like a major change in the association after negotiation, but then somehow it ends up being between 3.2 and 3.4 percent increase per life-month gained? ("Life-months gained", btw, is not a good phrase to use when talking about PFS, only OS.)**

We appreciate the editor for this insightful comment. In Figure 3, we presented the raw data of all indications supported by randomized controlled trials, illustrating the unadjusted relationship between treatment costs and survival benefits. We did not account for data distribution or exclude extreme outliers in this figure. However, during regression analyses (Table 2), we excluded outliers and log-transformed treatment costs based on data distribution. This explains the observed difference between Figure 3 and Table 2. The same explanation applies to Figure 4 and Table 3.

To address the editor's concern, we included both unadjusted and adjusted associations between treatment costs and clinical value for indications in Figures 3 and 4 in the revised manuscript. We also provided an explanation of the data processing steps for presenting the adjusted association in the Figure legends (*pages 20-21 in revised-track version*).

Furthermore, in response to the editor's comment, we have replaced the term 'life-months gained' with 'survival benefits' in the revised manuscript.

**I also couldn't follow the y axis on Fig 5. I also think the authors should report the general statistics on OS/PFS/QoL or ORR in the Results before showing the changes over time, so that readers can put the changes in context.**

We appreciate this constructive suggestion from the editor. In response, we have provided more detailed descriptions and explanations for Figure 5 by revising both the label and the caption (*page 22 in revised-track version*). Additionally, as recommended, we have included general statistics on OS, PFS, QoL, and ORR in the results section (*lines 279-288, page 9, and lines 303-304, page 10 in revised-track version*).

**I also had some trouble with the Discussion -- how does this study provide guidance for other countries negotiating prices?** **The authors don't describe much on how negotiation occurred, only that it did occur and the outcomes of it. Can the authors estimate how much savings to the health care system overall were achieved given the spending on the drugs in the study?**

We sincerely thank the editor for this constructive comment. To provide a more detailed context of price negotiation in China, we have added additional information in the supporting information *(S1 Text)*. The information we provided elaborated the experience of price negotiation from China, including who has been implementing the price negotiation, how the price negotiation has been implemented, and what accomplishments have been achieved through price negotiation. We hope such information could provide readers with a deeper understanding of price negotiation in China, thereby elucidating the policy implications of our study for other nations that are implementing or planning to implement price negotiation.

We appreciate the editor’s interest in the overall savings to the health care system achieved through price negotiation. While we acknowledge the importance of this aspect, it falls beyond the scope of our current research. Currently, we lack the necessary data on the utilization of these anticancer drugs for estimating overall savings in China. Nevertheless, the editor's insightful question has provided valuable guidance for our future research, for which we are sincerely grateful. We look forward to collecting data and conducting research on this matter in the future.

**Also, aren't there some potential concerns about the finding that there's greater reduction in prices for drugs** **offering the most clinical benefits? That seems like it works against private profit incentives to generate those most-valuable drugs.**

We apologize for any confusion, and we have revised relevant expressions to enhance clarity. We did not report the findings that there was greater reduction in prices for drugs offering the most clinical benefits. Instead, we found that the implementation of price negotiation has resulted in the reduction in the ‘variation of drug treatment costs for a given value’, with greater reductions observed for higher values *(lines 325-332,* *pages 24-26 in revised-track version*). In other words, for drugs with the same clinical value, the treatment costs have become more centralized after price negotiation. This means the implementation of price negotiation in China has led to drug pricing being better aligned with clinical value, especially for drugs with higher value.

We hope the editor would be satisfied with our revision.

Reply to Editors’ Comments for "Impact of price negotiation on the pricing of anticancer drugs in China" (PMEDICINE-D-23-02974R1)

We appreciate all the editors for the valuable and helpful comments on our manuscript. We have revised our manuscript based on these comments, highlighting the associated changes in red in the revised manuscript. The following responses address the comments in the order of occurrence.

**TITLE**

**Please revise your title according to PLOS Medicine's style. Your title must be nondeclarative and not a question. It should begin with main concept if possible. "Effect of" should be used only if causality can be inferred, i.e., for an RCT. Please place the study design ("A randomized controlled trial," "****An observational study," "A modelling study," etc.) in the subtitle (i.e., after a colon).**

In response to this comment, we have changed our title to 'Price negotiation and pricing of anticancer drugs in China: An observational study' and made relevant changes in the manuscript as the title changed.

**ABSTRACT**

**Please structure your abstract using the PLOS Medicine headings (Background, Methods and Findings, Conclusions).**

We have revised the abstract accordingly.

**Please combine the Methods and Findings sections into one section, “Methods and findings”.**

We have revised the abstract accordingly.

**Abstract Background: Provide the context of why the study is important. The final sentence should clearly state the study question.**

We have revised the abstract accordingly.

**Abstract Methods and Findings:**

**Please ensure that all numbers presented in the abstract are present and identical to numbers presented in the main manuscript text.**

We have confirmed that all numbers presented in the abstract are present and identical to numbers presented in the main manuscript text.

**Please include the study design, population and setting, the total number of indications you report on (103), and please explicitly state the main outcome measures.**

We have revised the abstract accordingly.

**Please include (brief) details of where and how you extracted relevant data to inform your study.**

We have revised the abstract accordingly.

**It might be helpful to include examples of the indications that you refer to and perhaps the different drug classes.**

We have provided examples of the cancer types intended to be treated by the indications, aligning with the results presented in Table 1 of the manuscript.

**Please quantify the main results with 95% CIs as well as p values. When reporting p values please report as <0.001 and where higher as p=0.002, for example. When reporting 95% CIs please separate upper and lower bounds with commas instead of hyphens as the latter can be confused with reporting of negative values.**

As suggested, we have quantified the main results regarding regression analyses with 95% CIs as well as p values.

**Please include any important dependent variables that are adjusted for in the analyses.**

We have included the dependent variables in the abstract.

**In the last sentence of the Abstract Methods and Findings section, please describe the main limitation(s) of the study's methodology.**

We have described the main limitations of our study’s methodology.

**Abstract Conclusions:**

**The opening sentence is currently very long and not very accessible (line 47, in particular). As written, it’s difficult to fully appreciate the implications of your study. Please revise in mind of the below points:**

*** Please address the study implications without overreaching what can be concluded from the data; the phrase "In this study, we observed ..." may be useful.**

*** Please interpret the study based on the results presented in the abstract, emphasizing what is new without overstating your conclusions.**

*** Please avoid vague statements such as "these results have major implications for policy/clinical care".  Mention only specific implications substantiated by the results.**

*** Please avoid assertions of primacy ("We report for the first time....")**

We have revised the conclusions accordingly.

**AUTHOR SUMMARY**

**At this stage, we ask that you include a short, non-technical Author Summary of your research to make findings accessible to a wide audience that includes both scientists and non-scientists. The authors summary should consist of 2-3 succinct bullet points under each of the following headings:**

**• Why Was This Study Done? Authors should reflect on what was known about the topic before the research was published and why the research was needed.**

**• What Did the Researchers Do and Find? Authors should briefly describe the study design that was used and the study’s major findings. Do include the headline numbers from the study, such as the sample size and key findings.**

**•What Do These Findings Mean? Authors should reflect on the new knowledge generated by the research and the implications for practice, research, policy, or public health. Authors should also consider how the interpretation of the study’s findings may be affected by the study limitations. In the final bullet point of ‘What Do These Findings Mean?’, please describe the main limitations of the study in non-technical language.**

**The Author Summary should immediately follow the Abstract in your revised manuscript. This text is subject to editorial change and should be distinct from the scientific abstract. Please see our author guidelines for more information: https://journals.plos.org/plosmedicine/s/revising-your-manuscript#loc-author-summary**

We have included the author summary following the abstract.

**INTRODUCTION**

**If there has been a systematic review of the evidence related to your study (or you have conducted one), please refer to and reference that review and indicate whether it supports the need for your study.**

We did not identify or conduct a systematic review of the evidence related to our study, and therefore, we were unable to refer to one.

**Line 104 – the term ‘more aligned’ is a little vague please revise for clarity.**

We thank the editors for this helpful comment and we have changed ‘more aligned’ to ‘better aligned’.

**METHODS and RESULTS**

**Line 110 – please (re)define NDRL here for the reader.**

We have redefined NRDL as suggested.

**Please clearly justify the start date of 2017 in the methods section. Throughout, please ensure** **all abbreviations are defined at first use for the reader.**

The selection of 2017 as the initial year corresponds to the initiation of price negotiation. We have explained the rationale for this selection in the revised manuscript. Additionally, we have ensured that all abbreviations are defined upon their first use.

**Please ensure that outcome measures are quantified with 95% CIs and p values. When reporting p values please report as <0.001 and where higher the exact p values a 0.002, for example.**

We have revised accordingly.

**In the manuscript text, please ensure that you indicate:**

**(1) the** **specific hypotheses you intended to test,**

We have ensured that the specific hypothesis is stated in the manuscript.

**(2) the** **analytical methods by which you planned to test them,**

We have ensured that the analytical methods planned to test the hypothesis are stated in the manuscript.

**(3) the analyses you actually performed, and**

We have confirmed that we performed and stated the planned analytical methods.

**(4) when reported analyses differ from those that were planned, transparent explanations for differences that affect the reliability of the study's results. If a reported analysis was performed based on an interesting but unanticipated pattern in the data, please be clear that the analysis was data-driven.**

This may not apply to our study, as the reported analyses are those we planned.

**TABLES**

**Please ensure that titles and captions are supplied for all tables (including those in Supporting Information files) which clearly describe the table content without the need to refer to the text.**

We have ensured that titles and captions are provided for all tables, and we have revised the table titles to provide clearer descriptions of their content.

**Please ensure that all abbreviations including those used for statistical reporting are defined the caption or an appropriate footnote.**

We have confirmed that all abbreviations were defined appropriately.

**Throughout, please indicate whether your analyses are adjusted or unadjusted and where you present adjusted analyses, please clearly detail the factors which have been adjusted for. To help facilitate transparent data reporting please also present the unadjusted analyses for comparison.**

We thank the editors for this constructive comment. We have made relevant revisions in the figures and the main manuscript, showing and indicating both the unadjusted analyses and the adjusted analyses.

For the regression analyses presented in all tables, we have clearly indicated in the notes section that treatment costs (the dependent variable) were adjusted by log-transforming and/or excluding the extreme outlier(s). As suggested, we have reported the unadjusted analyses in the Figures, while the analyses in the tables were all adjusted.

**Throughout, when reporting outcome measures please quantify with 95% CIs and p values. Please report as <0.001 and where higher the exact p value as 0.002, for example. Please avoid the use of asterisks to denote significance levels. Please use commas to separate upper and lower bounds of CIs.**

We have revised accordingly.

**Please see the academic editor comments above regarding your tables which we agree with. Please revise for improved clarity as requested.**

We have revised Tables 2 and 3, along with relevant tables in the supporting information, to enhance clarity.

**Table 1 – what does ‘origin country’ refer to in this context?**

We appreciate the editors for raising this question. In response, we have replaced the term 'origin country' with 'domestically developed' to enhance clarity. By using this term, we aim to convey whether the drug was developed within China or imported from abroad.

**Table 2 & 3 – we agree that these are hard to follow, the column headers are also uninformative. Please revise in mind of the aforementioned comments.**

We have revised the column headers and made other necessary revisions to enhance clarity.

**FIGURES**

**Please see the academic editor comments (above) regarding your figures which we agree with. Please revise as requested.**

In response to the comments of the academic editor and the editors, we have revised the figures by providing the unadjusted association and adjusted association simultaneously. We have also provided details on how we adjusted the association in caption.

**Please ensure that** **titles and captions are supplied for all figures (including those in Supporting Information files).**

We have ensured that titles and captions are supplied for all figures.

**Please ensure that all abbreviations including those used for statistical reporting are defined the caption or an appropriate footnote.**

We have checked and ensured that abbreviations were defined appropriately.

**Throughout, please quantify main outcome measures with 95% CIs and p values. Please report p as <0.001 and where higher the exact p value as 0.002, for example. Please use commas as opposed to hyphens to separate upper and lower CI bounds as the latter can be confused with reporting of negative values.**

We have revised accordingly for the main manuscript and tables.

**To help facilitate transparent data reporting please justify any deviations from the above.**

We have explained the deviations in the main manuscript and in the figure captions.

**Figures 3 and 4 – please present the adjusted results. For the purpose of transparent data reporting the unadjusted results can be presented for comparison.**

We have revised the figures by providing the unadjusted association and adjusted association simultaneously. We have also provided details on how we adjusted the association in caption.

**Figure 5 – we agree that the axis and labels are confusing, please revise**

We have revised the labels as well as the caption to give more detailed descriptions or explanations.

**DISCUSSION**

**Please also see the comments from the academic editor (above) regarding your discussion which we agree with. Please include additional discussion of these specific points.**

We thank the editors and the academic editor for the comments on the discussion. Since the academic editor suggests that we don't describe much on how negotiation occurred, we have provided such information in the appendix. We hope such information could provide readers with a deeper understanding of price negotiation in China, thereby elucidating the policy implications of our study for other nations that are implementing or planning to implement price negotiation.

**Opening paragraph, line 284 onwards – as for the abstract conclusions this is a little difficult to follow and for the more general reader could be particularly difficult. Suggest revising for improved clarity/accessibility.**

Following the suggestion, we have revised the expression to enhance clarity.

**Please present and organize the Discussion as follows: a short, clear summary of the article's findings; what the study adds to existing research and where and why the results may differ from previous research; strengths and limitations of the study; implications and next steps for research, clinical practice, and/or public policy; one-paragraph conclusion. Please refrain from the use of sub-headings such that the discussion reads as continuous prose.**

We have checked and presented the discussion following the instructions. We have also deleted the sub-heading we previously used.

**REFERENCES**

**For in-text reference callouts please place citations in square parentheses separate by commas. For example, [1,3,6] or [1-3]. Please check and amend throughout all sub-sections of the manuscript and supporting files.**

We have checked and amended accordingly.

**In the bibliography, please ensure that you list up to but no more than 6 author names followed by et al.**

We have checked and amended accordingly.

**For all web references please ensure you include an, ‘Accessed [date].’**

We have checked and amended accordingly.

**Journal name abbreviations should be those listed in the National Center for Biotechnology Information (NCBI) databases.**

We have checked and amended accordingly.

**SUPPORTING INFORMATION**

**Please cite your Supporting Information as outlined here:** [**https://journals.plos.org/plosmedicine/s/supporting-information**](https://journals.plos.org/plosmedicine/s/supporting-information)

We have revised accordingly.

**In the published article, supporting information files are accessed only through a hyperlink attached to the captions. For this reason, you must list captions at the end of your manuscript file. You may include a caption within the supporting information file itself, as long as that caption is also provided in the manuscript file. Do not submit a separate caption file.**

We have listed captions at the end of the manuscript file.

**Tables S4-S9 – these are similarly difficult to follow as those in the main manuscript. Please revise in mind of the comments detailed above and to ensure consistency of reporting throughout your manuscript.**

We have revised these Tables in accordance with the relevant comments provided for those Tables in the main manuscript.

We hope the editors would find our revision satisfactory.

Reply to Reviewers’ Comments for "Impact of price negotiation on the pricing of anticancer drugs in China" (PMEDICINE-D-23-02974R1)

We sincerely appreciate all the reviewers for carefully evaluating our revised manuscript, considering our responses to previous comments, and offering valuable new insights. In response, we have made necessary revisions, with the associated changes highlighted in red throughout the revised manuscript. The following section addresses each comment in the order of occurrence.

**Reviewer #1: In general, the authors have thoughtfully addressed the majority of my comments. The manuscript has seen significant improvement. I have one minor suggestion related to my third comment, which the authors may want to consider:**

**While some products have entered medical insurance negotiations in China based on conditional approval, we should still provide some discussion on this matter. It is advisable to suggest that the national medical insurance department should have distinct pricing policies for products that have not yet demonstrated clinical benefits. Alternatively, we can explore how other countries handle medical insurance policies for conditionally approved products to consider how we should proceed.**

We appreciate the referee for this insightful comment. As recommended, we have suggested that extra attention could be paid to addressing the uncertainty of clinical evidence or accounting for the strength of clinical evidence when setting pricing strategies in China, following our discussion about conditional approval (*lines 358-360, page 11 in revised-track version*).

**Reviewer #2: The authors had explained all the questions reasonably and revised the corresponding sections. The manuscript is proposed for publication.**

We sincerely thank the referee for reviewing our revised manuscript and responses to previous comments. The referee's proposal is greatly appreciated.

**Reviewer #3: This study assessed the impact of China's annual price negotiations on anticancer drugs, implemented since 2017, focusing on their cost and clinical value. It included indications of anticancer drugs negotiated from 2017 to 2022, analyzing clinical value data from pivotal trials (covering safety, survival, quality of life, and response rates) and calculating treatment costs. Using multivariate regression, the study examined the relationship between drug costs and clinical value pre- and post-negotiation. Results revealed a significant reduction in median treatment costs after negotiations, from $34,460.72 to $13,688.79. Initially, higher treatment costs correlated with greater clinical value. Post-negotiation, this correlation was less significant, though the variation in costs for given clinical values decreased. The study concludes that price negotiations in China effectively reduced drug prices while maintaining a correlation with clinical value, offering insights for price regulation in countries with limited resources and rising drug expenditures. Overall, this is a well-designed and conducted study. While it centers on China, its findings have potential applicability and value for a broader international audience.**

**Upon thorough examination, it's clear that the authors have effectively incorporated the valuable feedback from the four reviewers. The revisions have enhanced the paper substantially, and I find that it now aligns with the publication's standards. With no additional comments beyond what the reviewers have provided, I recommend accepting the revised manuscript in its current state for publication.**

We sincerely thank the referee for taking the time to review both our revised manuscript and our previous responses to the four reviewers. The referee's recommendation is greatly appreciated.
